# The SecA2 pathway of *Mycobacterium tuberculosis* exports effectors that work in concert to arrest phagosome and autophagosome maturation

**DOI:** 10.1371/journal.ppat.1007011

**Published:** 2018-04-30

**Authors:** Katelyn E. Zulauf, Jonathan Tabb Sullivan, Miriam Braunstein

**Affiliations:** Department of Microbiology and Immunology, University of North Carolina at Chapel Hill, Chapel Hill, North Carolina, United States of America; McGill UniversityHealth Centre, CANADA

## Abstract

To subvert host defenses, *Mycobacterium tuberculosis (Mtb)* avoids being delivered to degradative phagolysosomes in macrophages by arresting the normal host process of phagosome maturation. Phagosome maturation arrest by *Mtb* involves multiple effectors and much remains unknown about this important aspect of *Mtb* pathogenesis. The SecA2 dependent protein export system is required for phagosome maturation arrest and consequently growth of *Mtb* in macrophages. To better understand the role of the SecA2 pathway in phagosome maturation arrest, we identified two effectors exported by SecA2 that contribute to this process: the phosphatase SapM and the kinase PknG. Then, utilizing the *secA2* mutant of *Mtb* as a platform to study effector functions, we identified specific steps in phagosome maturation inhibited by SapM and/or PknG. By identifying a histidine residue that is essential for SapM phosphatase activity, we confirmed for the first time that the phosphatase activity of SapM is required for its effects on phagosome maturation in macrophages. We further demonstrated that SecA2 export of SapM and PknG contributes to the ability of *Mtb* to replicate in macrophages. Finally, we extended our understanding of the SecA2 pathway, SapM, and PknG by revealing that their contribution goes beyond preventing *Mtb* delivery to mature phagolysosomes and includes inhibiting *Mtb* delivery to autophagolysosomes. Together, our results revealed SapM and PknG to be two effectors exported by the SecA2 pathway of *Mtb* with distinct as well as cumulative effects on phagosome and autophagosome maturation. Our results further reveal that *Mtb* must have additional mechanisms of limiting acidification of the phagosome, beyond inhibiting recruitment of the V-ATPase proton pump to the phagosome, and they indicate differences between effects of *Mtb* on phagosome and autophagosome maturation.

## Author summary

*Mycobacterium tuberculosis (Mtb*) is the infectious agent of the disease tuberculosis. Inside the host, *Mtb* replicates primarily within the phagosome of macrophages. To replicate within macrophages, *Mtb* modifies the phagosome by inhibiting the normal host process of phagosomes maturing into acidified degradative phagolysosomes. In order to arrest this process of phagosome maturation, *Mtb* exports multiple effectors to the host-pathogen interface. Here we found that the specialized SecA2 protein export pathway of *Mtb* exports two such effectors: SapM and PknG. We discovered that SapM and PknG play non-redundant functions in phagosome maturation arrest by *Mtb*. We further demonstrated that SecA2 export of both SapM and PknG contributes to the ability of *Mtb* to replicate in macrophages. We also identified a role for the SecA2 pathway, SapM and PknG in arresting the host process of autophagosome maturation. Our research highlights how two effectors, SapM and PknG, work in concert but also have distinct roles in phagosome and autophagosome maturation arrest by *Mtb*.

## Introduction

In 2015, 1.8 million deaths were attributed to infection with *Mycobacterium tuberculosis* (*Mtb*), the causative agent of tuberculosis [1]. *Mtb* is an intracellular pathogen that subverts multiple antimicrobial mechanisms of the host in order to survive and replicate in macrophages [2]. To avoid trafficking to the antimicrobial environment of acidified phagolysosomes, *Mtb* blocks the normal series of phagosome maturation events that occurs following phagocytosis [2,3].

As a result, *Mtb* resides in phagosomes that resemble early endosomes in retaining Rab5 on their surface, avoiding host factors that drive downstream maturation events (e.g. phosphatidylinositol-3-phosphate [PI3P], Rab7, and the vacuolar-H^+^-ATPase [V-ATPase]) and failing to fuse with degradative lysosomes [4–7]. Notably, *Mtb* prevents phagosome recruitment and assembly of V-ATPase, a proton pump that acidifies the phagosome, which helps explain the failure of mycobacterial phagosomes to fully acidify [4,8,9].

Phagosome maturation is a complex multi-step process and there are multiple *Mtb* protein and lipid effectors that are thought to play a role in arresting phagosome maturation [10]. However, the specific function(s) of effectors and the interplay between effectors remains to be determined. It also remains unclear if all the effectors of this process are known. The gaps in our understanding are partly due to redundancy among effectors and the potential for effectors to have functions in other aspects of *Mtb* pathogenesis or physiology beyond phagosome maturation arrest [8,11–16]. These features of effectors make it difficult to study the contribution of individual effectors to phagosome maturation arrest using loss of function mutants.

In addition to residing in phagosomes, intracellular *Mtb* can also localize to double membrane bound compartments known as autophagosomes. Autophagosomes progress through similar maturation stages as phagosomes and culminate in fusion with lysosomes to form degradative autophagolysosomes [17]. As with phagosomes, *Mtb* is able to arrest autophagosome maturation and prevent fusion with lysosomes [18,19]. However, unlike the process of phagosome maturation arrest, there has been very little study of *Mtb* mechanisms and effectors of autophagosome maturation arrest.

Most of the reported effectors of *Mtb* phagosome maturation arrest are either exported to the bacterial cell wall or fully secreted [20]. In *Mtb*, the SecA2 protein export pathway is required for phagosome maturation arrest, which indicates that this pathway exports effectors required to inhibit phagosome maturation [21]. Unlike the paralogous SecA1 ATPase, which is responsible for the bulk of housekeeping export and is essential for bacterial viability, SecA2 is a non-essential specialized SecA ATPase required for exporting a relatively small subset of proteins [22–25]. Although not required for growth during *in vitro* broth culture, SecA2 is required for *Mtb* replication in macrophages and mice [23,26]. Unlike wild type *Mtb*, during macrophage infection, a *secA2* mutant of *Mtb* is delivered to acidified mature phagosomes [21]. The failure of the *secA2* mutant to arrest phagosome maturation is previously shown to be responsible for its intracellular growth defect [21].

We hypothesized that the role of the SecA2 pathway in phagosome maturation arrest is to export multiple effectors of the process. Here, we identify for the first time SapM, a secreted phosphatase previously reported to function in phagosome maturation arrest, as being exported by the *Mtb* SecA2 pathway [7,27]. We further show that the SecA2 dependent export of this protein contributes to both phagosome maturation arrest and intracellular growth of *Mtb*. By identifying a histidine residue that is essential for SapM phosphatase activity, we confirm that the phosphatase activity of SapM is required for its function. Along with SapM, our data indicates the existence of other SecA2-dependent effectors of phagosome maturation arrest and we identify the *Mtb* eukaryotic-like serine/threonine protein kinase PknG as one of these additional factors. By restoring export of SapM and PknG individually and in combination to the *secA2* mutant, we provide unique insight into specific steps in phagosome maturation arrest that are impacted by one or both of these effectors, as well as extend our understanding of the role of SecA2, SapM, and PknG to *Mtb* inhibition of autophagosome maturation. These studies additionally reveal the value of using the *secA2* mutant as a platform to study functions of effectors in phagosome maturation arrest.

## Results

### The SapM phosphatase is secreted by the SecA2 pathway

**Fig 1:**
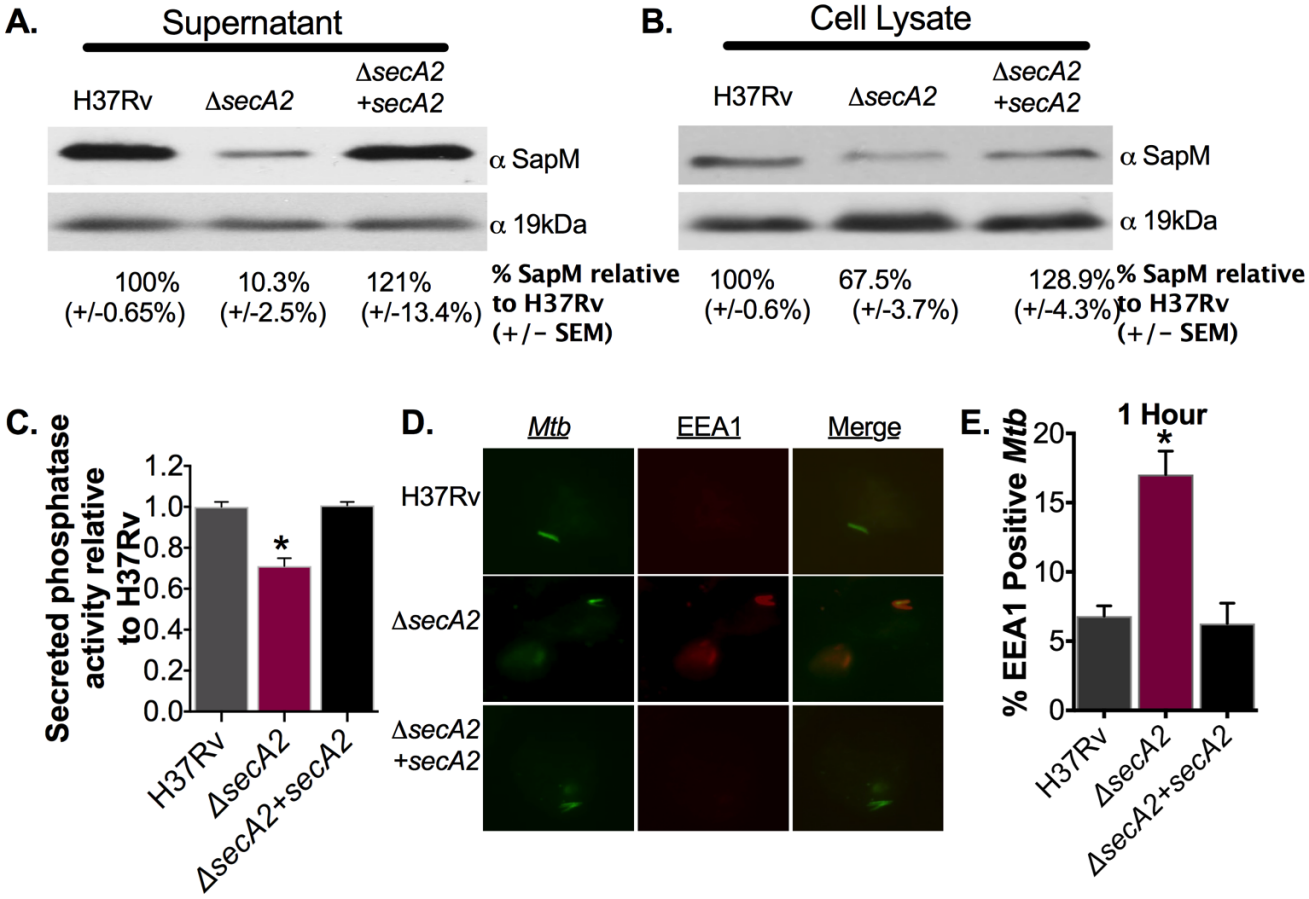
SapM export is dependent upon the SecA2 pathway. (A) Equal protein from culture supernatants or (B) cell lysates from the wild-type strain H37Rv, the *secA2* mutant and the complemented strain were examined for levels of SapM or the SecA2 independent loading control 19kDa by Immunoblot. Densitometry of triplicate blots was used to quantify the percentage of SapM levels (+/- SEM) in fractions relative to H37Rv (ImageJ). (C) Phosphatase activity in triplicate culture supernatant samples was examined by quantifying cleavage of pNPP. Rates of pNPP cleavage were normalized to H37Rv. (D) Quadruplicate wells of murine bone marrow derived macrophages were infected and EEA1 recruitment to phagosomes was assessed by Immunofluorescence. Representative images of EEA1 stained *Mtb* infected macrophages are shown. (E) The percentage of *Mtb* containing phagosomes that co-localized with EEA1 at 1hr post infection was determined. *p<0.01 ANOVA Holm-Sidak post Hoc test. Data represents at least two independent experiments.

With the goal of understanding the contribution of SecA2 to phagosome maturation arrest by *Mtb*, we tested the possibility that the SapM phosphatase is exported by the SecA2 pathway. SapM is a known effector of phagosome maturation arrest, [27]. Immunoblot analysis with SapM antisera was performed on *Mtb* culture supernatants. Compared to the parental *Mtb* strain, H37Rv, and a complemented strain, the *Mtb secA2* mutant had significantly reduced levels of secreted SapM, although a low residual level of SapM secretion was always observed in the mutant (Fig 1A). The amount of SapM in whole cell lysates was also reduced, albeit more modestly (Fig 1B). These reduced levels of SapM were not due to transcriptional effects in the *secA2* mutant, as shown by qRT-PCR measurements of *sapM* transcript in the *secA2* mutant compared to H37Rv in both broth cultures as well as in *Mtb* infected macrophages (S1A and S1B Fig). Thus, the lower levels of secreted SapM in the *secA2* mutant is the likely consequence of a SapM export defect, and the reduced cellular levels may be due to cytoplasmic SapM being unstable in the absence of export.

We also examined the contribution of SecA2 to SapM export by quantifying phosphatase activity in culture supernatants using p-nitrophenyl phosphate (pNPP) as a substrate. Even though this phosphatase assay is not specific for SapM (*i*.*e*. other phosphatases are detected), we observed less phosphatase activity in culture supernatants of the *secA2* mutant when compared to H37Rv or the complemented strain (Fig 1C). Importantly, in the presence of sodium molybdate, a known inhibitor of SapM, the secreted phosphatase activity of the *secA2* mutant was equivalent to that of H37Rv and complemented strains which is consistent with the *secA2* mutant being defective in SapM secretion. (S1C Fig) [28]. Together, the immunoblot and activity data provide the first evidence of SapM being secreted by the SecA2 pathway.

### SapM secretion by the SecA2 pathway limits EEA1 localization to phagosomes

SapM was previously shown using *in vitro* approaches to dephosphorylate PI3P, which should limit recruitment of PI3P binding proteins, such as EEA1, that promote downstream phagosome maturation events [7,29,30]. Consequently, we hypothesized that SapM secretion by the SecA2 pathway contributes to phagosome maturation arrest by enabling *Mtb* to avoid EEA1 localization to phagosomes. As a first step to test this possibility, murine bone marrow derived macrophages were infected with the *secA2* mutant, H37Rv or the complemented strain and EEA1 localization to *Mtb*-containing phagosomes was determined using the endogenous auto-fluorescent signal of *Mtb* and immunostaining with anti-EEA1 antibodies. Compared to phagosomes containing H37Rv or the complemented strain, which avoid EEA1 localization, phagosomes containing the *secA2* mutant exhibited significantly higher EEA1 co-localization at both 1hr and 24hrs post infection (*i*.*e*. time following a 4hr period of initial uptake/infection) (Fig 1D and 1E, S1D Fig).

**Fig 2:**
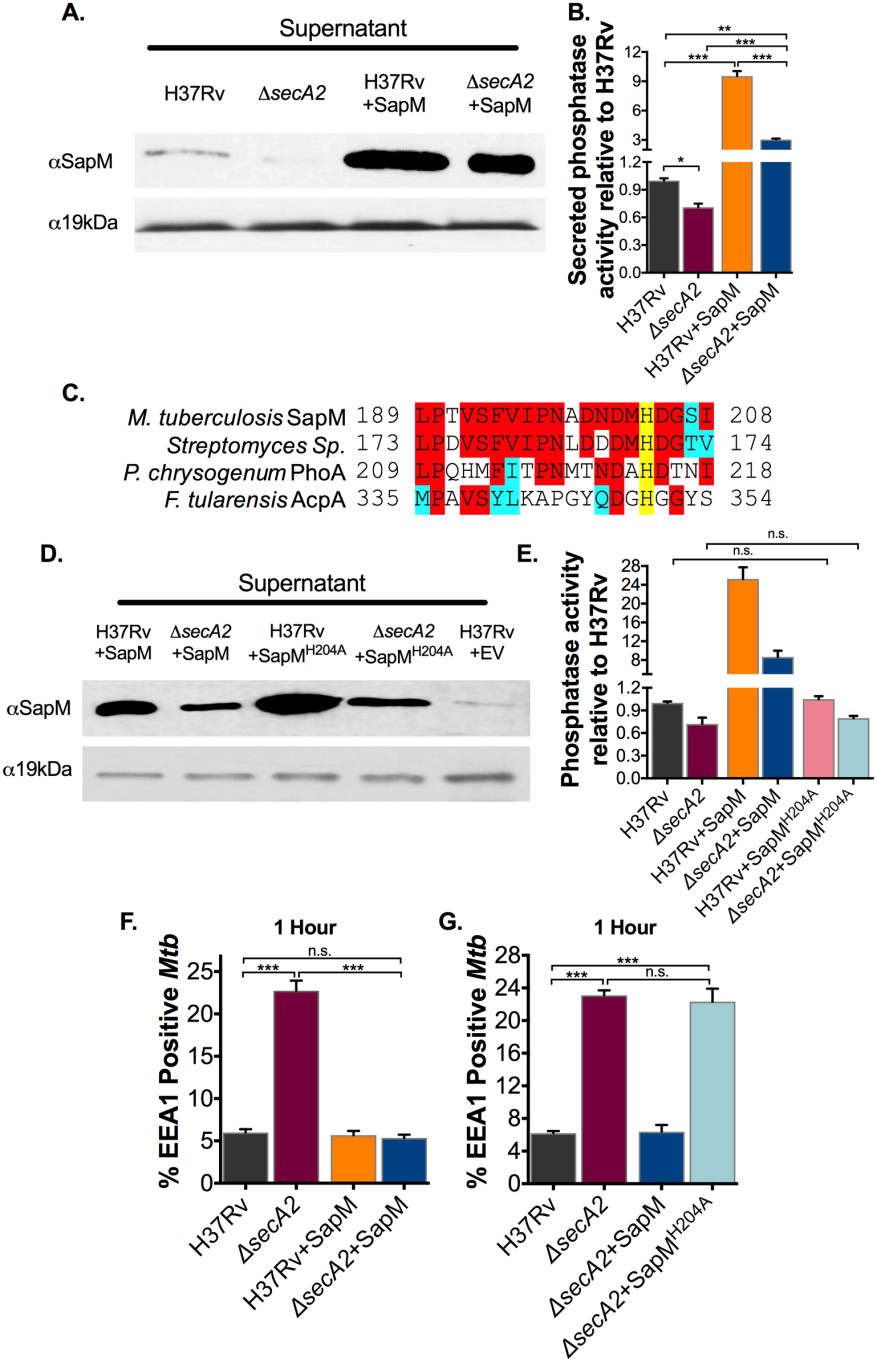
SecA2 secretion of SapM is required for EEA1 exclusion from *Mtb* containing phagosomes. (A) Equal protein from culture supernatants isolated from the H37Rv, the *secA2* mutant and the SapM overexpression strains was examined for levels of SapM or the SecA2 independent loading control 19kDa by Immunoblot. (B) Phosphatase activity of triplicate culture supernatant samples was examined by quantifying cleavage of pNPP. Rates of pNPP cleavage were normalized to H37Rv. (C) The potential active site of SapM was aligned to the amino acid sequence of an acid phosphatase from *Streptomyces sp*. (WP_063837857.1), PhoA from *Penicillium chrysogenum* and AcpA from *Francisella tulerenensis*. Identical residues are shaded red and similar residues are shaded blue. The conserved active Histidine is highlighted in yellow. (D) Equal protein from culture supernatants isolated from H37Rv and the *secA2* mutant overexpressing wild-type SapM as well as from H37Rv and the *secA2* mutant overexpressing SapM^H204A^ was examined for levels of SapM or the SecA2 independent loading control 19kDa by Immunoblot. (E) Phosphatase activity of triplicate culture supernatant samples was examined by quantifying cleavage of pNPP. Rates of pNPP cleavage were normalized to H37Rv. (F and G) The percentage of *Mtb* containing phagosomes that contain EEA1 was assessed in quadruplicate wells of *Mtb* infected BMDM by Immunofluorescence at 1hr post-infection. *p<0.05 **p<0.001 ***p<0.0001 ANOVA Holm-Sidak post Hoc test. Data represents at least two independent experiments.

We next set out to determine if the failure of the *secA2* mutant to prevent EEA1 recruitment to phagosomes is due to the SapM secretion defect of the mutant. For this purpose, we built a strain of the *secA2* mutant with the amount of secreted SapM restored to wild type levels. If SapM is the only SecA2-dependent effector preventing EEA1 recruitment, then restoring SapM secretion to wild type levels in the *secA2* mutant background should rescue this step of phagosome maturation arrest. However, if additional SecA2-dependent effectors exist with roles in this step of phagosome maturation arrest, their export will remain compromised and the EEA1 defect will persist. To restore the level of SapM secretion, we introduced a plasmid that overexpressed SapM in the *secA2* mutant background (*secA2*+SapM). In this *secA2* mutant strain, the level of secreted SapM was restored, even surpassing the level seen with H37Rv (Fig 2A). While the mechanism of restored secretion is not clear, we suspect the overexpressed SapM is exported by an alternate pathway, as some SapM is observed in culture supernatants of the *secA2* mutant (Fig 1A). Importantly, the overexpressed SapM was functional as demonstrated by the increased secreted phosphatase activity of the *secA2*+SapM strain (Fig 2B).

Using this *secA2*+SapM strain, we tested how restored SapM secretion affects EEA1 recruitment to *secA2* mutant containing phagosomes. Restored SapM secretion in the *secA2* mutant fully rescued the *secA2* mutant defect in preventing EEA1 (Fig 2F, S2A Fig) (*i*.*e*. the percent EEA1+ *Mtb* containing phagosomes was equivalent between *secA2*+SapM and H37Rv). This result indicates that the defect in SapM secretion of the *secA2* mutant accounts for the failure to exclude EEA1 from phagosomes. In other words, SecA2 secretion of SapM is required for *Mtb* to prevent EEA1 recruitment to phagosomes. The effect of overexpressing SapM was specific to the *secA2* mutant, as SapM overexpression in H37Rv did not further reduce EEA1 recruitment (Fig 2F, S2A Fig).

### SapM phosphatase activity prevents phagosomal EEA1 localization

Past studies lead to a model of SapM functioning to block phagosome maturation by dephosphorylating PI3P [7]. However, there is no direct evidence that the role of SapM in phagosome maturation arrest is through its phosphatase activity. By overexpressing a SapM variant lacking phosphatase activity in the *secA2* mutant we tested the significance of SapM phosphatase activity during macrophage infection. Catalytic residues and the active site of SapM have yet to be studied. To create a phosphatase defective SapM, we substituted an alanine for histidine 204, which aligns with a catalytically important residue in fungal acid phosphatases (Fig 2C) [31]. When plasmids overexpressing SapM or SapM ^H204A^ were introduced in the *secA2* mutant, the level of secreted SapM was comparable, as measured by immunoblot (Fig 2D). However, unlike overexpressed wild-type SapM, when SapM ^H204A^ was overexpressed there was no increase in secreted phosphatase activity, indicating H204 is essential for SapM phosphatase activity (Fig 2E). Using SapM ^H204A^, we then tested the importance of phosphatase activity to the role of SapM in preventing EEA1 recruitment to *Mtb* containing phagosomes. Unlike overexpressed SapM (*secA2*+SapM), SapM^H204A^ (*secA2*+SapM^H204A^) was unable to rescue the defect of the *secA2* mutant in preventing EEA1 recruitment (Fig 2G, S2B Fig). This result proves that the phosphatase activity of SapM is essential for SapM to exclude EEA1 from *Mtb* containing phagosomes.

### SapM affects multiple steps of phagosome maturation and is not the only SecA2-dependent effector of phagosome maturation arrest

**Fig 3:**
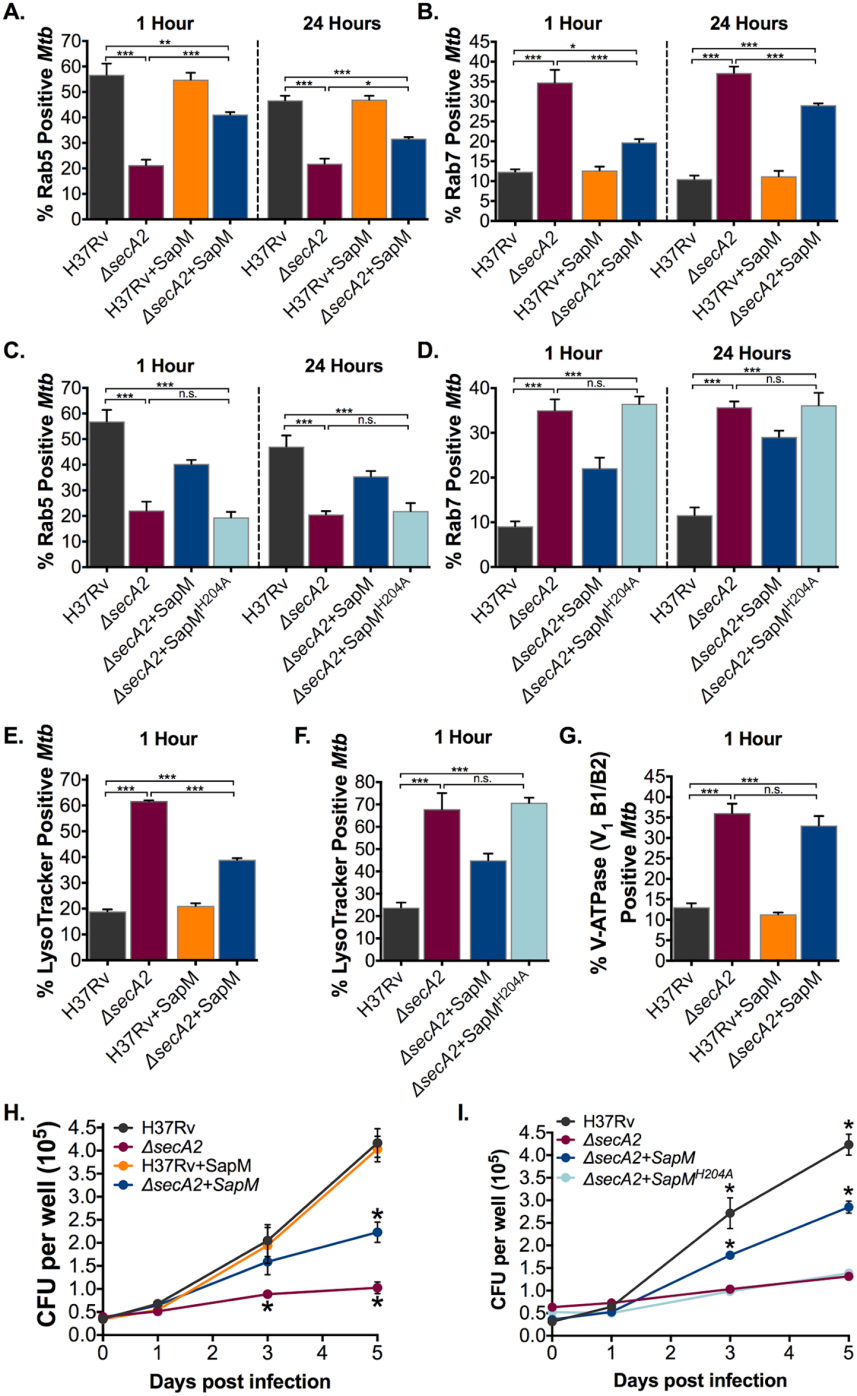
SecA2 secretion of SapM contributes to phagosome maturation arrest and intracellular growth. The percentage of *Mtb* containing phagosomes that contain (A, C) Rab5 and (B,D) Rab7 was assessed in quadruplicate wells of *Mtb* infected BMDM by Immunofluorescence at both 1hr and 24hrs post-infection. (E,F) The percentage of *Mtb* phagosomes that were acidified was determined using LysoTracker staining of quadruplicate wells of infected cells at 1hr post infection. (G) The percentage of *Mtb* containing phagosomes that contain V-ATPase was assessed in quadruplicate wells of *Mtb* infected BMDM by Immunofluorescence at 1hr post infection. (H,I) Triplicate wells of BMDM were infected at an MOI of 1 and CFU burden was assessed over the course of a 5 day infection. *p<0.05 **p<0.001 ***p<0.0001 ANOVA Holm-Sidak post Hoc test. Data represents at least two independent experiments.

During the normal process of phagosome maturation, Rab5 is recruited to early phagosomes and is then exchanged for Rab7 as phagosomes mature. However, *Mtb* has the effect of retaining Rab5 and excluding Rab7 from phagosomes [5] Using immunofluorescence microscopy, we measured percent co-localization of Rab5 and Rab7 with *secA2* mutant containing phagosomes. In contrast to H37Rv-containing phagosomes, *secA2* mutant-containing phagosomes retained less Rab5 and recruited more Rab7, confirming the *secA2* mutant is defective for phagosome maturation arrest (Fig 3A and 3B). Taking advantage of the *secA2*+SapM strain, we tested if SapM additionally impacts Rab5-Rab7 exchange. When secreted SapM was restored to the *secA2* mutant, a partial, but significant, rescue of *Mtb* inhibition of Rab5-Rab7 exchange on phagosomes was observed (*i*.e. restoring secreted SapM significantly increased Rab5 retention and reduced Rab7 recruitment) (Fig 3A and 3B). Furthermore, as shown with the phosphatase defective SapM^H204A^, the phosphatase activity of SapM is required for its function in inhibiting Rab5-Rab7 exchange (Fig 3C and 3D). However, because the *secA2*+SapM strain did not restore the block in Rab5-Rab7 exchange to levels seen with H37Rv infected macrophages, this data argues for the existence of additional *Mtb* effectors exported by the SecA2 pathway impacting this step of phagosome maturation. It is noteworthy that the effect of the *secA2*+SapM strain on Rab5 retention and Rab7 exclusion waned as infection progressed (1 hr versus 24 hrs post infection) (Fig 3A and 3B).

Avoiding phagosome acidification is another feature of *Mtb* phagosome maturation arrest that is impaired in *secA2* mutant containing phagosomes [21]. Using LysoTracker, an acidotropic dye that accumulates in acidified compartments, we examined if restoring secreted SapM to the *secA2* mutant rescues the ability of the mutant to avoid acidified phagosomes. The *secA2*+SapM strain was associated with a significant reduction in the percent LysoTracker co-localization (acidification) when compared to the *secA2* mutant, indicating that SapM secretion by the SecA2 pathway contributes to *Mtb* inhibition of phagosome acidification (Fig 3E, S3A Fig). However, the percentage of LysoTracker co-localization observed for the *secA2*+SapM strain was still significantly higher than that observed for H37Rv-containing phagosomes. This partial rescue reinforces the above conclusion that SapM is not the only SecA2-dependent effector of phagosome maturation arrest. The phosphatase activity of SapM is also required to prevent phagosome acidification as shown with SapM^H204A^ (Fig 3F, S3B Fig). We next examined the effect of restoring secreted SapM to the *secA2* mutant on the ability to inhibit V-ATPase, the proton pump complex that acidifies the phagosome [4]. We previously showed that V-ATPase is excluded from *Mtb* containing phagosomes but has a significantly higher association with *secA2* mutant-containing phagosomes [21]. In stark contrast to the effect restoring SapM secretion to the *secA2* mutant had on phagosome acidification, no effect was observed on recruitment of the V-ATPase V_1_B1/B2 subunits (Fig 3G, S3C Fig). To validate this result, we repeated the immunostaining utilizing antibodies that recognize a different component of the V-ATPase complex (V_0_a1). Again, the result revealed no effect of SapM on V-ATPase recruitment (S4A and S4B Fig). These results are significant in revealing a role of SapM in preventing phagosome acidification that is independent from inhibiting recruitment of V-ATPase to phagosomes.

### SapM contributes to the role of SecA2 in promoting *Mtb* intracellular growth

Having previously linked the failure of the *secA2* mutant to arrest phagosome maturation with the intracellular growth defect of the mutant, we tested the effect of restoring secreted SapM to the *secA2* mutant on growth in macrophages. Intracellular growth was monitored over time by plating macrophage lysates for viable bacilli. While there was no difference in bacterial burden 24 hrs post infection, significantly fewer *secA2* mutant bacilli were recovered after three and five days of infection compared to H37Rv (Fig 3H). When secreted SapM was added back to the *secA2* mutant, intracellular growth of the mutant significantly improved (Fig 3H). The improvement in intracellular growth was dependent on phosphatase activity of SapM, as SapM^H204A^ had no effect on intracellular growth of the *secA2* mutant (Fig 3I). However, intracellular growth of the *secA2*+SapM strain was not restored to the level exhibited by H37Rv, which reinforces the above conclusions that additional SecA2 exported effectors must exist. There was no effect on intracellular growth with SapM overexpression in H37Rv.

### SecA2 export of PknG contributes to phagosome maturation arrest and growth in macrophages

**Fig 4:**
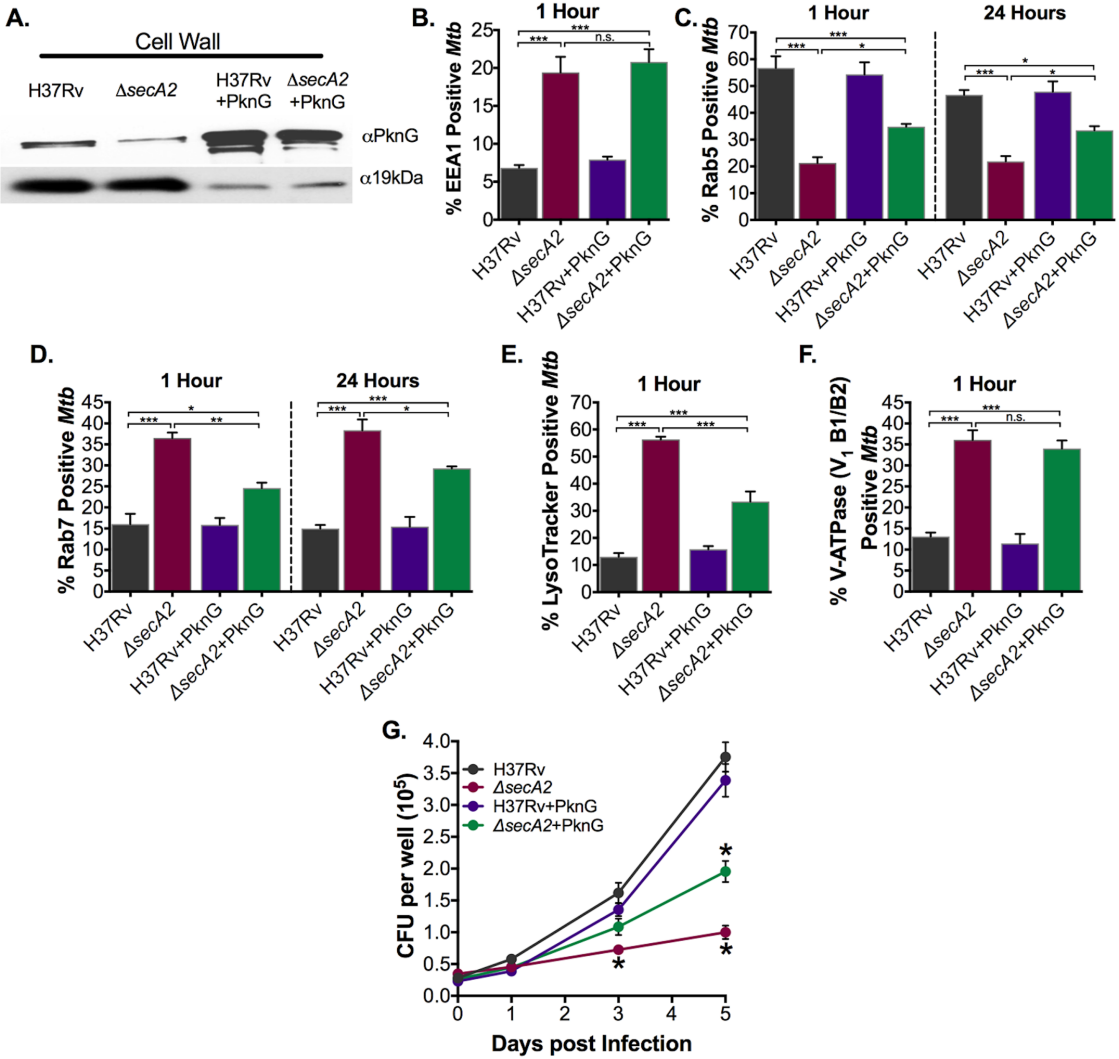
SecA2 export of PknG contributes to phagosome maturation arrest and growth in macrophages. (A) Cell wall fractions were isolated from *Mtb* strains and levels of PknG and the SecA2 independent loading control 19kDa were assessed by Immunoblot. 10x protein was loaded for H37Rv and *secA2* mutant than the corresponding *pknG* overexpression strains. (B) The percentage of *Mtb* containing phagosomes that contain EEA1 was assessed in quadruplicate wells of *Mtb* infected BMDM by Immunofluorescence at 1hr post-infection. The percentage of *Mtb* containing phagosomes that contain (C) Rab5 and (D) Rab7 was assessed in quadruplicate wells of *Mtb* infected BMDM by Immunofluorescence at both 1hr and 24hrs post-infection. (E) The percentage of *Mtb* phagosomes that were acidified was determined using LysoTracker staining of quadruplicate wells of infected cells at 1hr post infection. (F) The percentage of *Mtb* containing phagosomes that contain V-ATPase was assessed in quadruplicate wells of *Mtb* infected BMDM by Immunofluorescence at 1hr post-infection. (G) Triplicate wells of BMDM were infected at an MOI of 1 and CFU burden was assessed over the course of a 5 day infection. *p<0.05 **p<0.001 ***p<0.0001 ANOVA Holm-Sidak post Hoc test. Data represents at least two independent experiments.

Recent studies identified the PknG kinase, a protein with functions in *Mtb* physiology as well as phagosome maturation arrest, as being exported by the SecA2 pathway to the cell wall of *Mtb* and *Mycobacterium marinum* [13,24,32,33]. To elucidate the role of SecA2 export of PknG in phagosome maturation arrest, we took the same approach as used with SapM of testing the effect of restoring PknG export to the *secA2* mutant. By overexpressing *pknG* in the *secA2* mutant (*secA2*+PknG) we were able to restore export of PknG to greater than wild type levels (Fig 4A).

In contrast to the full rescue in EEA1 inhibition observed with SapM restoration in the *secA2* mutant, restoring PknG export to the *secA2* mutant had no effect on EEA1 (Fig 4B, S5A Fig). However, restoring exported PknG to the *secA2* mutant significantly increased the ability of the *secA2* mutant to retain Rab5 and exclude Rab7 on phagosomes (Fig 4C and 4D). This result reveals a role for PknG in preventing Rab5-Rab7 exchange that has not been described previously. However, like the *secA2*+SapM strain, the *secA2*+PknG strain was not as effective as H37Rv in inhibiting Rab5-Rab7 exchange, indicating it is not the only SecA2 exported effector involved in inhibiting this step of phagosome maturation. Intriguingly, unlike SapM, the effect seen with PknG restoration was consistent at both 1hr and 24hrs post infection (Fig 4C and 4D).

When we examined phagosome acidification using LysoTracker, restored PknG export in the *secA2* mutant partially rescued the ability of the mutant to inhibit phagosome acidification (Fig 4E, S5B Fig). However, as with restoring SapM secretion, recruitment of V-ATPase subunits V_1_B1/B2 and V_0_a1 were both unaffected by restoring export of PknG in the *secA2* mutant (Fig 4F, S4A, S4B and S5C Figs).

Finally, we tested the effect of restored levels of exported PknG on intracellular growth of the *secA2* mutant. The *secA2*+PknG strain grew significantly better than the *secA2* mutant in macrophages, indicating SecA2 export of PknG contributes to intracellular growth of *Mtb* but, again, additional SecA2 exported proteins are also required, as growth was not restored to the levels seen with H37Rv (Fig 4G).

### When added back simultaneously, SapM and PknG have a combined effect on phagosome maturation arrest and intracellular growth

**Fig 5:**
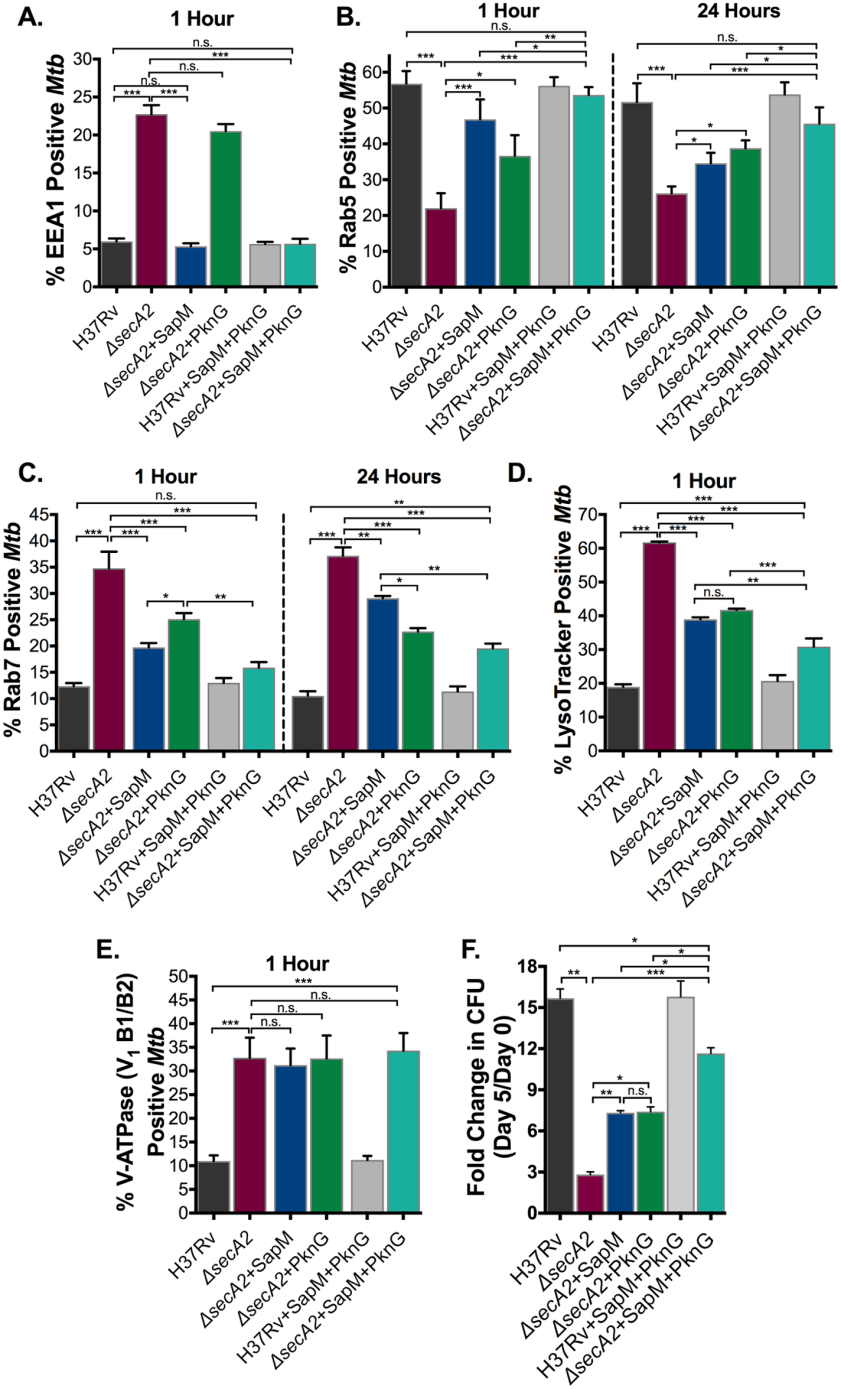
SecA2 export of both SapM and PknG contributes to phagosome maturation arrest and intracellular growth. (A) The percentage of *Mtb* containing phagosomes that contain EEA1 was assessed in quadruplicate wells of *Mtb* infected BMDM by Immunofluorescence at 1hr post-infection. The percentage of *Mtb* containing phagosomes that contain (B) Rab5 and (C) Rab7 was assessed in quadruplicate wells of *Mtb* infected BMDM by Immunofluorescence at both 1hr and 24hrs post-infection. (D) The percentage of *Mtb* phagosomes that were acidified was determined using LysoTracker staining of quadruplicate wells of infected cells at 1hr post-infection. (E) The percentage of *Mtb* containing phagosomes that contain V-ATPase subunit V_1_ B1/B2 was assessed in quadruplicate wells of *Mtb* infected BMDM by Immunofluorescence at 1hr post-infection. (F) Triplicate wells of BMDM were infected at an MOI of 1 and CFU burden was assessed over the course of a 5 day infection. The fold change in CFU over the course of the 5 day macrophage infection for each *Mtb* strain was calculated. *p<0.05 **p<0.001 ***p<0.0001 ANOVA Holm-Sidak post Hoc test. Data represents at least two independent experiments.

We next tested the effect of restoring export of SapM and PknG in combination to determine if these effectors have cumulative effects and if together they are sufficient to fully rescue the defects of a *secA2* mutant. We simultaneously overexpressed *sapM* and *pknG* to restore export of both effectors in the *secA2* mutant (*secA2*+SapM+PknG). As expected, the *secA2*+SapM+PknG strain fully inhibited EEA1 recruitment, like the *secA2*+SapM strain (Figs 2F and 5A, S6A Fig). When we examined Rab5 and Rab7 localization on phagosomes, simultaneous restoration of exported SapM and PknG to the *secA2* mutant inhibited Rab5-Rab7 exchange significantly more than restoration of either effector individually (Fig 5B and 5C). In fact, when compared to H37Rv at 1hr post infection, full rescue of the Rab5-Rab7 exchange inhibition was observed for the *secA2*+SapM+PknG strain. However, at 24hrs post infection the effect waned, which is reminiscent of what was observed with the *secA2*+SapM strain (Fig 3A and 3B).

In regard to phagosome acidification, the *secA2*+SapM+PknG strain had a greater effect on inhibiting phagosome acidification (LysoTracker) than observed with restoration of either effector individually (Fig 5D, S6B Fig). However, phagosome acidification was still not inhibited to wild-type levels by the *secA2*+SapM+PknG strain (Fig 5D, S6B Fig). Furthermore, even when export of both effectors was restored, exclusion of the V_1_B1/B2 or V_0_a1 subunits of V-ATPase was not rescued (Fig 5E, S4A, S4B and S6C Figs).

Finally, we tested the effect of restoring export of both effectors on growth of the *secA2* mutant in macrophages. The *secA2*+SapM+PknG strain grew significantly better than the *secA2* mutant with each effector restored individually (Fig 5F, S6D Fig). However, as seen with phagosome maturation arrest, the *secA2*+SapM+PknG strain was not fully rescued in its ability to grow intracellularly (Fig FG, S6D Fig).

Thus, the *secA2*+SapM+PknG strain revealed a cumulative effect of adding back exported SapM and PknG on Rab5-Rab7 exchange, acidification and intracellular growth. However, in nearly all cases adding back these two effectors was insufficient to restore phenotypes to the level seen with H37Rv, which indicates the existence of even more SecA2-dependent effectors of phagosome maturation arrest.

### Hv1 localization to phagosomes is inhibited by *Mtb* but does not depend on the SecA2 pathway

**Fig 6:**
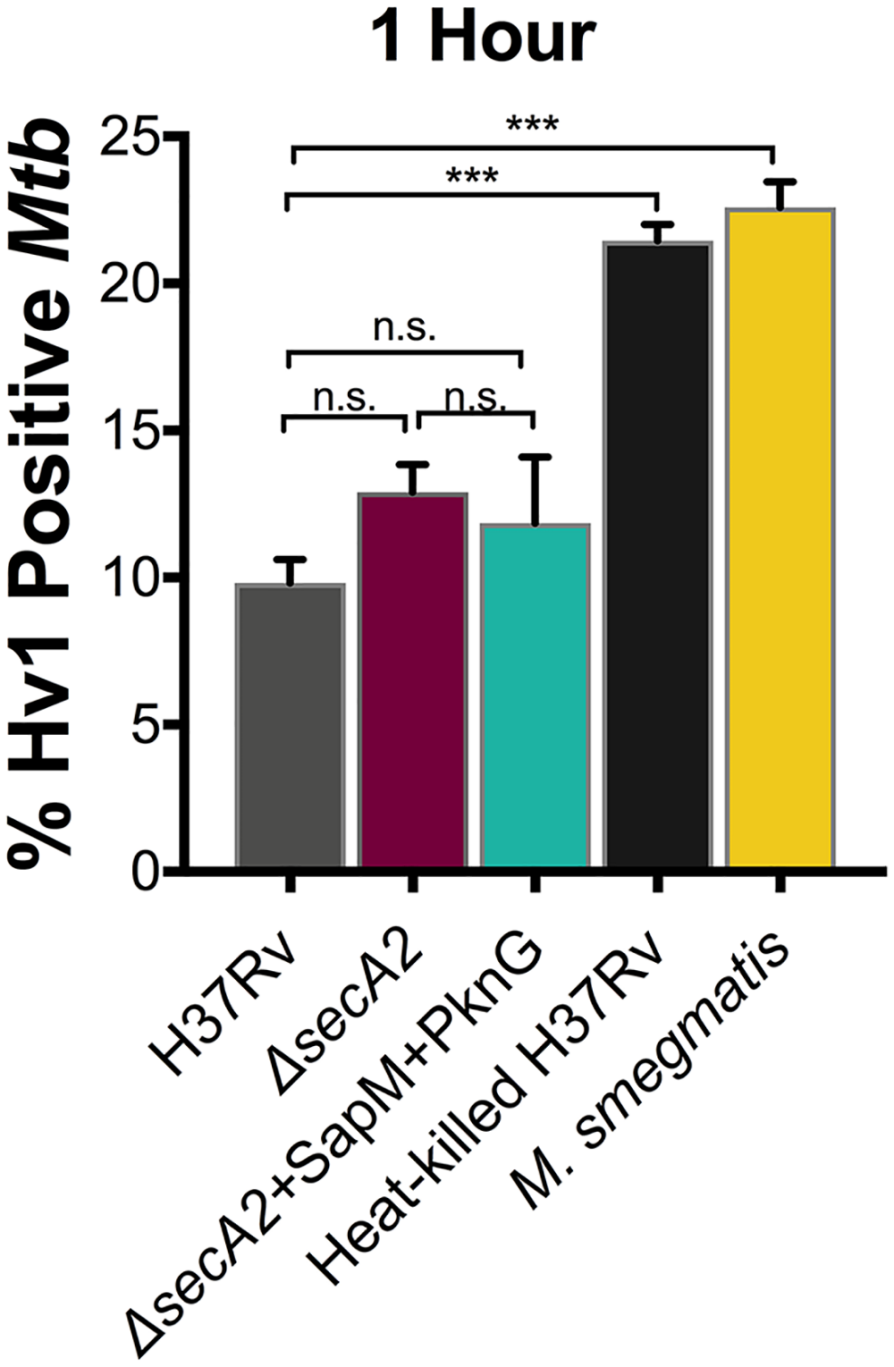
Mtb inhibits Hv1 recruitment to phagosomes. The percentage of *Mtb* containing phagosomes that contain Hv1 was assessed in quadruplicate wells of *Mtb* infected BMDM by immunofluorescence at 1hr post-infection. Heat-killed H37Rv and *M*. *smegmatis* were included in the infection as positive controls. ***p<0.0001 ANOVA Holm-Sidak post Hoc test. Data represents at least two independent experiments.

The discrepancy between the effects of SapM and PknG on phagosome acidification and V-ATPase localization suggests that *Mtb* has another mechanism of blocking acidification in addition to inhibiting V-ATPase recruitment. Hv1 is a voltage gated proton channel that was recently shown to localize to the phagosomal membrane of macrophages and to contribute to phagosome acidification along with V-ATPase [34,35]. Using immunofluorescence microscopy, we measured percent co-localization of Hv1 with H37Rv containing phagosomes. Only a small percentage (9.8%) of H37Rv localized to Hv1-positive phagosomes (Fig 6). In contrast to live H37Rv, the level of Hv1 co-localization was significantly higher in macrophages infected with either heat-killed H37Rv or non-pathogenic *Mycobacterium smegmatis* (21.4% and 22.5% respectively) (Fig 6). Thus, live *Mtb* has a lower association with Hv1-containing phagosomes than dead *Mtb* or non-pathogenic mycobacteria. However, there was no significant difference in Hv1 association in H37Rv versus *secA2* mutant infected macrophages (Fig 6). Therefore, while limiting Hv1 recruitment may be another mechanism by which live *Mtb* limits acidification of phagosomes, it does not account for the role of the SecA2 exported effectors SapM and PknG in this process.

### The SecA2 pathway is required to inhibit autophagosome maturation (flux)

**Fig 7:**
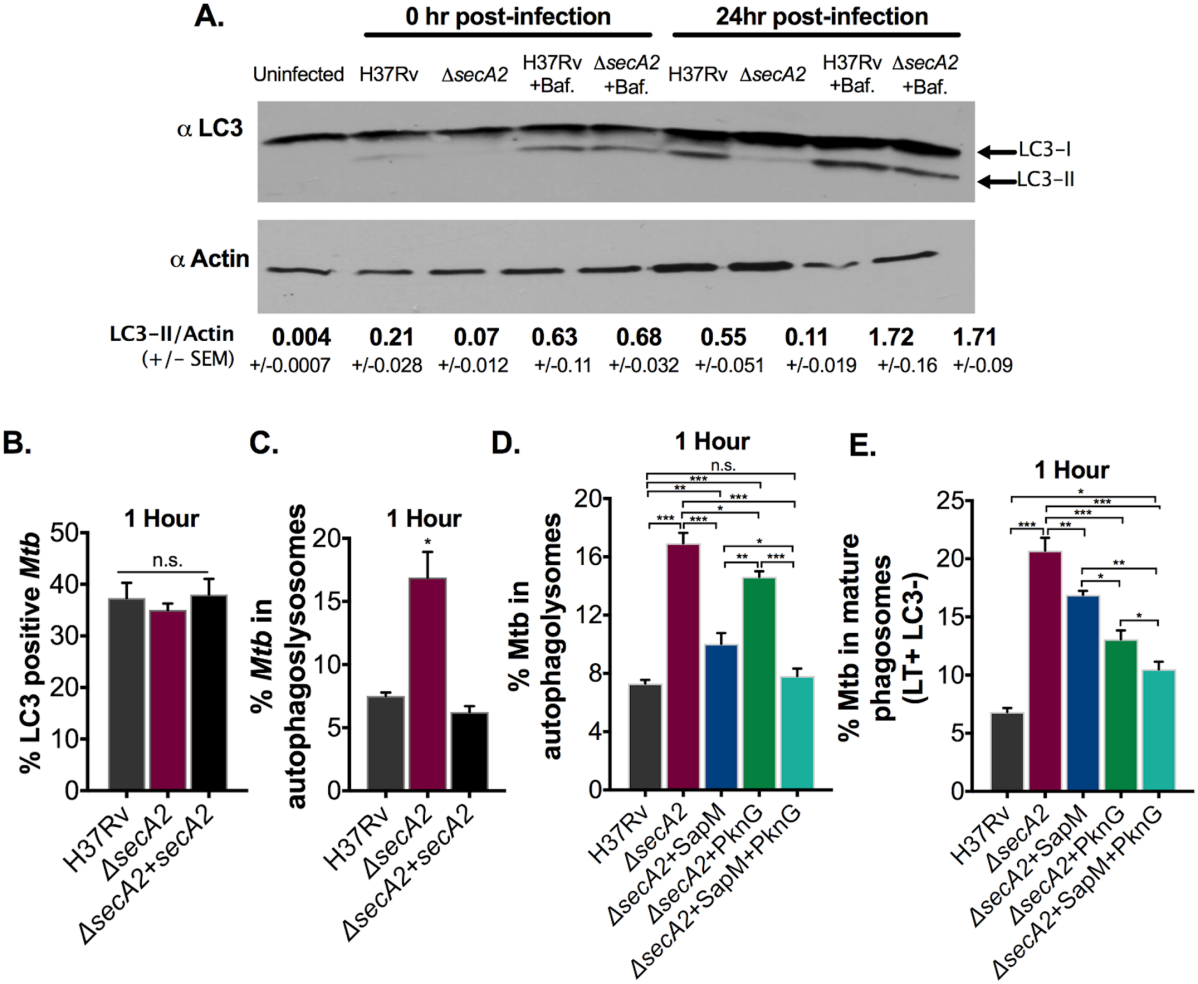
SecA2 is required for *Mtb* inhibition of autophagosome maturation. (A) RAW 264.7 cells were infected with H37Rv and the *secA2* mutant at an MOI of 10 and lysed either immediately after infection or 24hrs post-infection. One set of infected cells was treated with 100nm Bafilomycin A1 (Baf). Cell lysates were examined for levels of LC3 or the loading control Actin. Densitometry of triplicate blots was used to quantify LC3-II relative to Actin +/- SEM (ImageJ). Quadruplicate wells of RAW-Difluo mLC3 cells were infected with H37Rv, the *secA2* mutant and the complemented strain at an MOI of 1. (B) The percentage of LC3+ *Mtb* (RFP+) was assessed at 1hr post infection. (C) The percentage of *Mtb* that was localized in an autophagolysosome (RFP+GFP-) was assessed at 1hr post infection. Quadruplicate wells of RAW-Difluo mLC3 cells were infected with H37Rv and the *secA2* mutant SapM and/or PknG restoration strains at an MOI of 1. (D) The percentage of *Mtb* that was localized in an autophagolysosome (RFP+GFP-) was assessed at 1hr post infection. (E) The percentage of *Mtb* phagosomes that were acidified was determined using LysoTracker (LT) staining of quadruplicate wells of infected cells at 1hr post-infection. Mature phagosomes were identified by lack of LC3 (LC3-) and presence of LT staining (LT+RFP-). *p<0.05 **p<0.001 ***p<0.0001 ANOVA Holm-Sidak post Hoc test. Data represents at least two independent experiments.

In addition to phagosome maturation arrest, *Mtb* inhibits the maturation of autophagosomes to autophagolysosomes which is sometimes referred to as autophagy flux [18,19]. To determine if the SecA2 pathway is additionally required for autophagosome maturation arrest, we used LC3-II, the lipid modified form of LC3 that is associated with autophagosomes, to monitor autophagy in H37Rv and *secA2* mutant infected RAW 264.7 macrophages [36]. Lower levels of LC3-II were observed in *secA2* mutant infected macrophages when compared to H37Rv infected macrophages both immediately after the 4hr infection and 24 hrs post infection. (Fig 7A). The lower LC3-II levels were not due to a reduced bacterial burden in *secA2* mutant infected RAW cells as there was no difference in intracellular burden of H37Rv or the *secA2* mutant at these time points (S7E Fig). The lower levels of LC3-II in *secA2* mutant infected macrophages could indicate a defect of the *secA2* mutant in arresting autophagosome maturation such that there are more mature autophagosomes resulting in more LC3-II degradation. To test this possibility *Mtb* infected cells were treated with Bafilomycin A1, which blocks autophagosome acidification, maturation and the associated degradation of LC3-II. With Bafilomycin A1 treatment, the levels of LC3-II were comparable in *secA2* mutant and H37Rv infected macrophages. This result is consistent with the *secA2* mutant being defective in the ability to arrest autophagosome maturation (Fig 7A).

To further examine the requirement for the SecA2 pathway in autophagosome maturation arrest we utilized RAW 264.7 macrophages expressing a dual RFP::GFP::LC3 fusion protein (RAW-Difluo mLC3 cells). While RFP is resistant to the acidic environment of the autophagolysosome, GFP is acid sensitive and the signal is quenched in autophagolysosomes. By quantifying the number of RFP+ and GFP+/- autophagosomes, this cell line can report on autophagosome maturation. When we infected cells expressing RFP::GFP::LC3 with the *secA2* mutant, H37Rv or the complemented strain, there was no difference in the percent of *Mtb* that co-localized with any LC3+ compartments (RFP+, GFP+/-) (Fig 7B and S7A Fig). However, when we specifically examined the association of *Mtb* with mature autophagosomes by quantifying the percentage of *Mtb* that localize to autophagolysosomes (RFP+, GFP-), we found a significantly higher association of the *secA2* mutant with autophagolysosomes than either H37Rv or the complemented stain. Together, the LC3-II immunoblots and the RFP::GFP::LC3 reporter indicate an additional role of the SecA2 pathway in autophagosome maturation arrest (Fig 7C, S7B Fig).

### SecA2 export of SapM and PknG contributes to inhibition of autophagosome maturation (flux)

Using the *secA2* mutant strains with restored export of SapM and/or PknG, we next set out to determine if SecA2 export of SapM and PknG contributes to the function of SecA2 in autophagosome maturation arrest. Restored export of either SapM or PknG reduced localization of the *secA2* mutant in autophagolysosomes indicating both SapM and PknG contribute to *Mtb* inhibition of autophagosome maturation (Fig 7D, S8A Fig). Notably, restored SapM secretion resulted in a more significant reduction in *secA2* localization to autophagolysosomes than PknG (Fig 7D, S8A Fig). Simultaneous restoration of SapM and PknG export was more effective than restoration of either effector individually (Fig 7D, S8A Fig). In fact, when compared to H37Rv, full rescue of autophagosome maturation arrest was observed for the *secA2*+SapM+PknG strain (Fig 7D, S8A Fig).

A benefit of utilizing the RFP::GFP::LC3 expressing cell line is the ability to simultaneously examine autophagosome and phagosome maturation in the same cells. To monitor phagosome maturation, we quantified co-localization of LC3 negative (LC3-) *Mtb* with LysoTracker. In LC3- phagosomes, the *secA2* mutant localized more frequently to mature LysoTracker positive phagosomes than H37Rv (Fig 7E, S8B Fig). Moreover, the *secA2*+SapM and *secA2*+PknG strains exhibited significantly reduced localization to mature LC3- phagosomes when compared to the *secA2* mutant (Fig 7E, S8B Fig). Interestingly, unlike with autophagosome maturation arrest, the effect on phagosome maturation of adding back exported SapM to the *secA2* mutant was significantly less than that of PknG (Fig 7E, S8B Fig). The *secA2*+SapM+PknG strain exhibited even greater rescue of phagosome maturation arrest than observed with restoration of either effector individually (Fig 7E, S8B Fig). However, in contrast to autophagosome maturation, the *secA2*+SapM+PknG strain was not fully rescued in its ability to arrest phagosome maturation (Fig 7E, S8B Fig). The function of SapM in both autophagosome and phagosome maturation arrest depends on SapM phosphatase activity, as shown by the *secA2*+SapM^H204A^ strain remaining defective in both processes (S8C and S8D Fig). Together, these results demonstrate that both phagosome and autophagosome maturation arrest depend on the SecA2 pathway, SapM, and PknG. However, these experiments also reveal differences in the contribution individual effectors make to each process and expose the existence of additional SecA2-dependent effectors that are required for phagosome maturation arrest but not necessarily for autophagosome maturation arrest.

## Discussion

Phagosome maturation arrest by *Mtb* is complex and much remains to be learned about the effectors involved in the process and how they work together. We showed previously that the SecA2 pathway is required for *Mtb* to inhibit phagosome maturation; however, the SecA2-dependent effectors of phagosome maturation arrest remained unknown [21]. Here, we identified two SecA2 exported effectors of *Mtb* phagosome maturation arrest as the phosphatase SapM and the kinase PknG. Then, using a strategy of adding back export of SapM and PknG to the *secA2* mutant, we not only established the significance of the role of the SecA2 pathway in exporting these proteins but we identified steps in phagosome maturation that are impacted by these factors individually and in combination. Moreover, we revealed that the SecA2 pathway, SapM, and PknG also function in inhibiting autophagosome maturation.

Prior to this study, SapM was not known to be secreted by the SecA2 export pathway. By testing the requirement for SecA2 in the export of a set of known effectors of phagosome maturation arrest (SapM, LpdC, Ndk) we identified SapM as a SecA2-exported protein [15,37]. The SecA2 pathway did not contribute to LpdC or Ndk secretion, and these effectors were not studied further (S9 Fig). PknG was identified as being exported by the SecA2 pathways of *Mtb* and *M*. *marinum* in recent proteomic studies [24,32]. While SapM and PknG are both known to function in phagosome maturation arrest and there are reports of *Mtb* mutants lacking these effectors having defects in phagosome maturation arrest, our understanding of their roles in inhibiting specific steps of phagosome maturation is far from complete [7,12,13,27].

We established the significance of SecA2 export of SapM and PknG to phagosome maturation arrest and intracellular growth, using the strategy of adding back export of these proteins to the *secA2* mutant. To create the necessary strains, we reasoned that overexpressing SecA2-dependent proteins in the *secA2* mutant could boost their export through the alternate mechanism, possibly the SecA1-dependent pathway, that accounts for the residual export in the *secA2* mutant of SapM, PknG and all SecA2 exported proteins identified to date [23,24,32,38]. Using overexpression, we achieved our goal of producing a *secA2* mutant strain that has at least as much exported SapM and/or PknG as detected in the wild type H37Rv strain. Notably, even when overexpressed, the *secA2* mutant exported less SapM and PknG than the corresponding H37Rv overexpression strain, confirming the dependency of these effectors on SecA2 for export. The effects of SapM and/or PknG overexpression were specific to the *secA2* mutant and specific to the overexpressed proteins. H37Rv was unaffected by increased levels of these proteins and equivalent levels of overexpressed SapM ^H204A^ in the *secA2* mutant had no effects. We also repeated the experiments using a single-copy vector with reduced, though still higher than wild-type levels, of secreted SapM and saw comparable restoration of phagosome maturation arrest and intracellular growth (S11 Fig).

The *secA2* mutant strains with restored levels of exported SapM and/or PknG allowed us to investigate steps in phagosome maturation affected by these effectors. Past studies demonstrate purified SapM can dephosphorylate PI3P *in vitro* [7]. This data led to a model for secreted SapM dephosphorylating PI3P and inhibiting recruitment of PI3P binding proteins, such as EEA1, to the phagosome to arrest phagosome maturation. However, critical details of this model have not been confirmed, including an effect of SapM on EEA1 and proof that SapM functions as a phosphatase to arrest phagosome maturation. Thus, our demonstration that restored levels of exported wild type SapM, but not the phosphatase defective SapM ^H204A^, inhibits EEA1 localization to phagosomes provides important validation of the model. Adding back wild type SapM also partially restored inhibition of Rab5-Rab7 exchange and this again depends on SapM phosphatase activity. This effect of SapM on Rab5-Rab7 exchange was not previously noted, but it is consistent with the role of PI3P in Rab5-Rab7 exchange and is intriguing given a report of SapM binding to Rab7 [39–41]. The SapM effect on Rab5-Rab7 exchange was reproducibly more extreme at 1hr versus 24hrs post infection (Fig 3A and 3B). The temporal nature of effector functions revealed by this data reveals another layer of complexity to phagosome maturation arrest by *Mtb*.

The strategy of overexpressing PknG to restore exported levels to the *secA2* mutant was used previously in *M*. *marinum* [32]. Similar to what we observed, adding back exported PknG to the *secA2* mutant of *M*. *marinum* results in a partial restoration of phagosome maturation arrest. However, in the *M*. *marinum* study, the effect of PknG was only assessed on co-localization with the late lysosomal-associated membrane protein (LAMP1) [32]. Since the function(s) of PknG that impact phagosome maturation is unknown, we took advantage of the *secA2*+PknG strain to reveal effects on individual steps of phagosome maturation. PknG had no effect on EEA1 recruitment to phagosomes, but it did partially restore inhibition of Rab5-Rab7 exchange, revealing for the first time a function of PknG in inhibiting Rab5-Rab7 exchange. When exported SapM and PknG were added back simultaneously, a combined effect was observed that resulted in complete inhibition of Rab5-Rab7 exchange at 1hr post infection but waned as infection progressed. Thus, multiple SecA2-dependent effectors act on the same step of phagosome maturation and the combinatorial effects of these effectors suggests SapM and PknG work through different but complementary mechanisms.

Studies of PknG function in phagosome maturation arrest are complicated by the fact that, in addition to being exported, PknG functions in the bacterial cytoplasm in glutamate metabolism, regulation of the TCA cycle, and in a redox homeostatic system (RHOCS) that contributes to resistance to oxidative stress [11,12,33,42]. Because *Mtb* mutants with RHOCS defects are delivered to mature phagosomes and have intracellular growth defects, it raised the possibility that the redox function of PknG explains its role in phagosome maturation arrest [12]. However, this does not appear to be the case as the *secA2* mutant did not exhibit a RHOCS defect, as assessed by sensitivity to redox stress, and overexpression of PknG did not increase resistance to redox stress (S10 Fig).

The ability of *Mtb* to exclude V-ATPase from the phagosome is generally assumed to account for the lack of acidification of *Mtb* phagosomes [4,43]. Therefore, our demonstration that restoration of SapM and/or PknG export to the *secA2* mutant partially rescued the acidification defect of the *secA2* mutant but had no effect on recruitment of V-ATPase was a surprise. These results indicate the existence of additional mechanism(s) for *Mtb* to prevent phagosome acidification that were not previously appreciated. One possibility is that *Mtb* affects an additional proton transporter that contributes to phagosome acidification in conjunction with V-ATPase, such as Hv1 [34,35]. Unlike the V-ATPase, nothing is known about how Hv1 interfaces with bacterial pathogens. Because Hv1 also impacts NADPH oxidase-dependent generation of reactive oxygen species (ROS) by providing a compensating charge for electrons transferred to superoxide, an effect on Hv1 phagosomal levels could potentially not only affect phagosome acidification but also ROS production [35,44,45]. Our data suggests that *Mtb* actively limits Hv1 recruitment to macrophages, but in a manner that is independent of SecA2, SapM and PknG.

Alternate explanations for how *Mtb* affects phagosome acidification independent of V-ATPase exclusion include inhibiting the counter ion flux that is required for acidification to occur or direct inhibition of the V-ATPase pump [46]. In terms of the latter possibility, phosphorylation of the ATPase subunit of the V-ATPase is known to regulate activity of the proton pump, which raises the possibility of SapM and/or PknG affecting acidification by impacting phosphorylation of the V-ATPase [47].

Finally, because adding back SapM and PknG failed to rescue the mutant defect in excluding V-ATPase from the phagosome, there is at least one additional SecA2-dependent effector involved in this step of phagosome maturation arrest. PtpA, which binds subunit H of V-ATPase and thereby excludes the proton pump from phagosomes, is a candidate for this missing SecA2-exported effector [8]. Unfortunately, our inability to detect secreted PtpA in *Mtb* cultures prevented us from determining if PtpA is secreted by the SecA2 pathway.

Compared to phagosome maturation arrest, even less is known about autophagosome maturation arrest by *Mtb*. Using the RFP::GFP::LC3 reporter, we were able to reveal a role for SecA2 export in the maturation arrest of both LC3- phagosomes and LC3+ autophagosomes. It is important to note in this study, we are unable to distinguish between autophagosomes and other LC3+ compartments, including LC3 associated phagosomes (LAP) so we cannot exclude a function for SecA2 in those processes. Using the *secA2*+SapM+PknG strain we were able to demonstrate a function for both SapM and PknG in autophagosome maturation arrest by *Mtb*. Our data confirms a recent study using *sapM* transfected cells that suggests a role for SapM in autophagosome maturation arrest but this is the first evidence indicating a function for PknG in this process [39].

Intriguingly, the SecA2 exported effectors do not affect both autophagosome and phagosome maturation equally. SapM seems to have more of an effect on autophagosomes while PknG is more impactful on altering phagosomes. Further, while simultaneous restoration of both effectors was able to fully rescue the defect of the *secA2* mutant in autophagosome maturation, it was not sufficient to rescue the defect in phagosome maturation. Our results highlight the overlap in *Mtb* factors involved in phagosome and autophagosome maturation but at the same time reveal differences in specificity of *Mtb* effectors for both processes.

It is important to note that by focusing on effectors exported by the SecA2 pathway, our study does not rule out or diminish the significance of effectors that are exported by other routes. However, at the same time, the fact that the *secA2* mutant exhibits phagosome and autophagosome maturation arrest defects indicates that SecA2-independent effectors are not sufficient on their own to block these critical macrophage responses.

When we investigated the effect of SecA2 export of SapM and PknG on *Mtb* growth in macrophages, we found adding back either effector individually improved intracellular growth of the *secA2* mutant while restoring export of both effectors simultaneously resulted in a further improvement. These results reinforce prior studies indicating that the role of SecA2 in inhibiting *Mtb* delivery to mature phagosomes is required for intracellular growth [21]. Furthermore, the effect of SecA2, SapM and PknG on phagosome maturation arrest will likely extend beyond promoting replication in macrophages. By arresting phagosome maturation, *Mtb* also limits the presentation of antigenic peptides to the immune system [48].

In summary, our studies demonstrate that multiple effectors require the SecA2 pathway for their export and function in phagosome maturation arrest and they provide unique insight into how *Mtb* effectors work in concert to inhibit phagosome and autophagosome maturation. Our studies also revealed the advantages of using of the *secA2* mutant as a platform to study the function of effectors individually or in combination. This approach provides an alternative to studying effectors through deletion analysis, which can be problematic for effectors that share redundant functions or for effectors that have additional unrelated functions in *Mtb* (such as PknG). In this study, we discovered new layers of complexity in how *Mtb* arrests phagosome maturation (multiple means of inhibiting acidification, temporal effects), exposed a new host factor inhibited by *Mtb* (Hv1), uncovered distinct and cumulative effects of a pair of effectors, and revealed a broad role of the SecA2 pathway in phagosome and autophagosome maturation arrest that involves SapM, PknG and additional effectors that await identification.

## Materials and methods

### Ethics statement

This study included the use of mice and followed recommendations in the Guide for the Care and Use of Laboratory Animals of the National Institutes of Health. The protocol was approved by the International Animal Care and Use Committee at the University of North Carolina at Chapel Hill (protocol: 15–018.0).

### Strains and media conditions

In this study we used the *Mycobacterium tuberculosis* wild type strain H37Rv, and the Δ*secA2* mutant (mc^2^3112) generated in the H37Rv background as well as the wild-type *Mycobacterium smegmatis* strain MC^2^155. [23]. The plasmids and strains over-expressing *sapM* and/or *pknG* constructed for this study are listed in S1 and S2 Tables respectively.

*Mtb* strains were cultured in either liquid Middlebrook 7H9(BD) or on solid Middlebrook 7H10(BD) or 7H11 (Sigma) media supplemented with 0.05% Tween 80, 0.5% glycerol, 1x albumin dextrose saline (ADS) and kanamycin (20μg/ml) or hygromycin (50μg/ml) when appropriate. Sauton media was used for preparation of culture supernatants containing 30mM DL-asparagine, 7mM sodium citrate, 3mM potassium phosphate dibasic, 4mM magnesium sulfate, 0.2mM ferric ammonium citrate and 4.8% glycerol adjusted to a pH of 7.4. For cell wall isolation, we utilized a modified Middleboook 7H9 based media containing 0.1% glycerol, 1mM proprionic acid, 0.1% tyloxapol, 0.1M MES (buffer), 0.5% BSA, and adjusted to a pH of 6.5 [24].

### SapM Site-directed mutagenesis

The Histidine at position 204 of SapM was changed to an Alanine using site directed mutagenesis to generate SapM^H204A^. The *sapM* expression plasmid pJTS130 was used as a template. Primer sequences are as follows: 5'-cgatcgagccgtcggccatgtcgttgtcgg-3' and 5'-ccgacaacgacatggccgacggctcgatcg-3'. Dpn1 (NEB) was added to degrade the methylated template. Mutation was confirmed by sequencing.

### Culture supernatant isolation

For culture supernatant isolation, cultures were first grown to log-phase in Middlebrook 7H9 with 0.05% tyloxapol. Cultures were then washed in Sauton media and grown in Sauton media with 0.05% tyloxapol for 5 days after which cultures were washed again to remove detergent and sub-cultured into 100ml Sauton media (no detergent) at a starting OD600 of 0.25 for 2 days. Then the entire 100 ml culture was centrifuged at 3500 rpm and supernatants were collected and double filtered with a 0.2μm filter to remove cells. Culture supernatants were concentrated 100-fold using a 15 ml capacity 10,000 MW cut off centrifugal filter (Millipore). For Immunoblots, proteins were isolated by precipitation using 10% trichloroacetic acid overnight. To confirm that the supernatants were free of cytosolic contamination due to cell lysis, samples were examined by Immunoblot for absence of the cytoplasmic mycobacterial proteins SigA, GroEL, and SecA1.

### Cell wall isolation

For cell wall isolation, *Mtb* was first grown in 7H9 0.05% Tyloxapol to mid-log phase and then sub-cultured into the modified 7H9 media at a starting OD600 of 0.125. Cultures were harvested when they reached an OD600 of 1.0 and were then sterilized by gamma-irradiation in a JL Shephard Mark I 137Cs irradiator (Dept. of Radiobiology, University of North Carolina at Chapel Hill) prior to removal from BSL-3 containment. Subcellular fractions were isolated as previously described [49]. Briefly, irradiated cells were suspended in 1X PBS containing protease inhibitors and lysed by passage four times through a French pressure cell. Unlysed cells were removed by centrifugation at 3500 rpm to generate clarified whole cell lysates (WCLs), which were then spun at 25,000 rpm for 30 minutes to pellet the cell wall fraction.

### Immunoblots

Protein concentrations were determined by Bicinchoninic acid assay (Pierce). Equal protein for whole cell lysates, cell wall fractions, or concentrated culture supernatants was run on a SDS-PAGE gel, and then transferred to nitrocellulose membranes. After transfer, the membranes were blocked for one hour and then probed with primary antibodies. Antibodies to *Mtb* proteins were kind gifts of Vojo Deretic, University of New Mexico (SapM), Zakaria Hmama, University of British Columbia (LpdC and NdkA), Yossef Av-Gay, University of British Columbia (PknG) and Douglas Young, Imperial College (19kDa). LC3 and Actin antibodies were acquired from Cell Signaling Technologies. Antibodies were used at the following dilutions (SapM 1:5,000, LpdC 1:2,000, NdkA 1:2,000, PknG 1:5,000, 19kDa 1:20,000, LC3 1:500, and Actin 1:1000) Secondary antibodies were conjugated to horseradish peroxidase (BioRad) and signal was detected using chemiluminescence (Western Lighting Perkin Elmer).

### Phosphatase activity assay

SapM phosphatase activity was assayed as described previously [28]. The phosphatase activity of 5 μg of culture supernatants was assessed for triplicate samples. Each reaction contained 0.1mM Tris base pH 6.8 and 50mM p-nitrophenyl phosphate (pNPP)(New England Biolabs) with either 2 mM sodium tartrate to inhibit background phosphatase activity or 1 mM sodium molybdate to inhibit SapM activity. Samples were incubated at 37°C and the absorbance at 405nm was measured every minute for two hours. We then calculated the rate of pNPP conversion and normalized the data to H37Rv.

### Quantitative Real-time PCR

To isolate RNA from *Mtb* grown in vitro, triplicate *Mtb* cultures were grown in modified 7H9 medium to an OD_600_ of 1.0 and pelleted by centrifugation at 3,000 rpm for 10 min. Bacteria were lysed in 1 ml 3:1 chloroform-methanol, then vortexed with 5 ml TRIzol (Invitrogen) and incubated for 10 min at room temperature. Phases were separated by centrifugation at 3,000 rpm for 15 min at 4°C, and RNA was precipitated from the upper phase using 1X volume of isopropanol. RNA was pelleted by centrifugation at 12,000 rpm for 30 min at 4°C, washed twice with cold 70% ethanol, and resuspended in RNase-free water.

Mycobacterial RNA was isolated from *Mtb* infected macrophages as previously described [50,51]. Triplicate plates containing 2x10^7^ RAW 264.7 cells were infected at an MOI of 10 for 4 hrs. After 24hrs of infection, cells were washed with PBS and lysed using a guanidine thiocyanate buffer as previously described [47]. *Mtb* was pelleted by centrifugation and resuspended in 65°C TRIzol. Glass beads were added to the samples and the samples were vortexed to maximize lysis. Chloroform was added for a final concentration of 20%. Phases were separated by centrifugation at 3,000 rpm for 15 min at 4°C, and RNA was precipitated from the upper phase using 1X volume of isopropanol. RNA was pelleted by centrifugation at 12,000 rpm for 30 min at 4°C, washed twice with cold 70% ethanol, and resuspended in RNase-free water.

RNA samples were treated with DNase (Promega) and then column purified (Zymo RNA clean and concentrator kit). Following RNA isolation, cDNA was synthesized with random primers using the iScript cDNA Synthesis Kit (BioRad). Real-time PCR was completed using 25ng of cDNA template in triplicate technical replicates using the SensiMix SYBR and fluorescein kit (Bioline). Transcripts were normalized to the housekeeping gene *sigA*. Primer sequences are for *sapM* (ATCGTTGCTGGCCTCATGG and AGGGAGCCGACTTGTTACC) and *sigA* (GAGATCGGCCAGGTCTACGGCGTG and CTGACATGGGGGCCCGCTACGTTG).

### Macrophage infections

For bone marrow-derived macrophages (BMDM), femurs were isolated from C57/Bl6 (Jackson Labs) mice and flushed with complete DMEM (DMEM [Sigma] supplemented with 10% Heat inactivated fetal bovine serum [FBS] 5mM non-essential amino acids and 5mM L-glutamine). Bone marrow cells were washed, re-suspended and plated in complete DMEM containing 20% L-929 cell conditioned media (LCM). After six days, the cells were lifted off the plates using cold 5mM EDTA in PBS. Macrophages were seeded at 2 × 10^5^ macrophages/well in complete DMEM containing 20% LCM using either eight-well chambered slides to monitor growth of *Mtb* or chambered cover slips for microscopy experiments. After resting 24 hours the macrophages were infected with *Mtb* grown to log-phase, and washed twice with PBS containing 0.05% Tween 80 and diluted in warm complete DMEM. BMDM were infected at an MOI of 1.0 for four hours. Infected macrophages were then washed three times with pre-warmed complete DMEM to remove extracellular bacteria. Macrophages were lysed using 0.1% Triton X-100 at various time points and lysates were plated for cfu determination or slides were fixed in 4% paraformaldehyde (PFA) for immunofluorescence staining The 1 hour (hr) and 24 hr post infection time points reflect time following 4 hours of initial uptake/infection. At both the 1 and 24hr time points there were no differences in intracellular viability for any of the strains used in this study (Figs 3 and 4, S6 Fig). *M*. *smegmatis* infections followed the same procedure as Mtb. To prepare heat-killed H37Rv, H37Rv was prepared for infection as described above but heated to 80°C for 1hr prior to dilution in complete DMEM.

RAW 264.7 cells were cultured in DMEM supplemented with 10% FBS. Cells were seeded at 1*10^6^ macrophages/well (6-well plate) or 1*10^5^ macrophages/well (8-well chamber slide). For immunoblots, RAW cells were infected at an MOI of 10 for 3 hrs and washed three times with pre-warmed DMEM to remove extracellular bacteria. Bafilomycin A1 (Sigma) was utilized at a concentration of 10nM and maintained throughout the course of the experiment. Cells were lysed using RIPA buffer (50mM Tris-HCL pH 7.4, 1% NP-40, 0.25% Sodium deoxycholate, 150mM NaCl, and protease inhibitors). For cfu determination RAW cells were infected at an MOI of 1 for 4 hrs and washed three times with pre-warmed DMEM to remove extracellular bacteria. Cells were lysed using 0.1% Triton X-100 at various time points and lysates were plated for cfu determination.

RAW-Difluo mLC3 Cells expressing RFP::GFP::LC3 (InvivoGen) were cultured in DMEM supplemented with 10% FBS and zeocin. Cells were seeded at 1*10^5^ macrophages/well without zeocin and infected at an MOI of 1 for 4 hrs and washed three times with pre-warmed DMEM to remove extracellular bacteria. At 1hr or 24hrs post infection cells were fixed in 4% PFA.

### LysoTracker staining and immunofluorescent microscopy

For LysoTracker staining, media on *Mtb* infected BMDM was replaced with prewarmed DMEM containing 100nM LysoTracker Red DND99 (Invitrogen) for BMDM and 100nM LysoTracker Deep Red (Invitrogen) for RAW 264.7 cells and incubated for one hour. After which, media was removed and the slides fixed in 4% PFA.

For immunofluorescence, media was removed from *Mtb* infected macrophages and the slides were submerged in 4% PFA. The fixed slides were submerged in PBS to remove residual PFA and then cells were permeabilized with 0.1% Triton-X 100 in PBS for 5 minutes at room temperature, washed in PBS and blocked in PBS containing 10% donkey serum. Antibodies to the mammalian markers Rab5 (S-19) [52], Rab7 (H-50) [52], V-ATPase B1/B2 (H-180) [8] and V-ATPase A1 (D-20) and Texas Red conjugated donkey anti-rabbit or donkey anti-goat secondary antibodies were acquired from Santa Cruz Biotechnology. Antibodies to EEA1 were acquired from Abcam (ab2900) [53]. Antibodies to Hv1 were acquired from Invitrogen (PA5-21008). Primary antibodies were used at a 1:50 dilution in PBS with 3% serum and incubated overnight at 4°C. After which slides were washed in PBS, and secondary antibodies conjugated to TR fluorophores were used at 1:100 dilution in PBS with 3% serum and incubated at room temperature for one hour. Slides were washed to remove the secondary antibody and Fluormount-G (Southern Biotech) was added to each well to protect the fluorescent signal. As controls we included uninfected cells and single or no antibody controls. Widefield fluorescence microscopy was performed using an Olympus IX-81 controlled by the Volocity software package. All images were taken using a 60X oil-immersion objective. To visualize *Mtb* in infected macrophages we used the endogenous autofluorescence of the bacteria. Mycobacterial autofluorescence was visualized using a CFP filter cube (Semroc) with an excitation band of 426-450nm and emission band of 467-600nm [21]. As the autofluorescent signal quenches quickly, samples were prepared in the dark and the CFP channel was the first visualized on the microscope. A minimum of ten fields per well were captured and a minimum of 250 bacteria per well were scored for phagosomal markers. For each experimental group four replicate wells (*i*.*e*. ≥1000 bacteria per infection condition) were analyzed per experiment and data represents a minimum of two independent experiments. Representative images for each phagosomal marker are shown in Supplemental S12–S19 Figs.

### Oxidative stress resistance

To assess sensitivity to oxidative stress *Mtb* cultures were exposed to 5mM H_2_O_2_ in 7H9+ADS 0.05% Tween 80 for 24 and 48 hours. Survival was assessed by plating for viable CFU. Cultures without H_2_O_2_ were included as controls. Strains tested in this manner include H37Rv and the *secA2* mutant with and without PknG overexpression. A *pstA1*::*tn* mutant (generous gift of Jyothi Rengarajan, Emory University) which is extremely sensitive to oxidative stress was included as a control [54,55].

## Supporting information

S1 Table Plasmids used in this study. (XLSX)

S2 Table Strains used in this study. (XLSX)

S1 Fig SapM is exported in a SecA2 dependent manner. (A)RNA was isolated from triplicate broth cultures of H37Rv and the *secA2* mutant and *sapM* transcript was quantified by RT-PCR. Transcripts were normalized to the housekeeping gene *sigA*. (B)RNA was isolated from triplicate samples of RAW 264.7 cells infected with either H37Rv or the *secA2* mutant and *sapM* transcript was quantified by RT-PCR. Transcripts were normalized to the housekeeping gene *sigA*. (C) Phosphatase activity in triplicate culture supernatant samples was examined by quantifying cleavage of pNPP in the presence of sodium molybdate. Rates of pNPP cleavage were normalized to H37Rv. (D) The percentage of *Mtb* containing phagosomes that contain EEA1 was assessed in quadruplicate wells of *Mtb* infected BMDM by Immunofluorescence at 24hrs post-infection. *p<0.001 ANOVA Holm-Sidak post Hoc test. Data represents at least two independent experiments.
(TIFF)

S2 Fig SapM phosphatase activity is required for EEA1 exclusion from *Mtb* phagosomes at 24 hours post-infection. (A and B) The percentage of *Mtb* containing phagosomes that contain EEA1 was assessed in quadruplicate wells of *Mtb* infected BMDM by Immunofluorescence at 24hrs post-infection. ***p<0.0001 ANOVA Holm-Sidak post Hoc test. Data represents at least two independent experiments.
(TIFF)

S3 Fig SecA2 export of SapM contributes to *Mtb* phagosome maturation arrest at 24 hours post-infection. (A and B) The percentage of *Mtb* phagosomes that were acidified was determined using LysoTracker staining of quadruplicate wells of infected cells at 24hrs post infection (C) The percentage of *Mtb* containing phagosomes that contain V-ATPase was assessed in quadruplicate wells of *Mtb* infected BMDM by Immunofluorescence at 24hrs post infection. ***p<0.0001 ANOVA Holm-Sidak post Hoc test. Data represents at least two independent experiments.
(TIFF)

S4 Fig SecA2 export of SapM and PknG does not contribute to inhibition of V-ATPase recruitment by *Mtb*. The percentage of *Mtb* containing phagosomes that contain V-ATPase subunit V_0_ a1 was assessed in quadruplicate wells of *Mtb* infected BMDM by Immunofluorescence at (A) 1hr and (B) 24 hours post-infection. *p<0.05 **p<0.001 ***p<0.0001 ANOVA Holm-Sidak post Hoc test. Data represents at least two independent experiments.
(TIFF)

S5 Fig SecA2 export of PknG contributes to *Mtb* phagosome maturation arrest at 24 hours post-infection. (A) The percentage of *Mtb* containing phagosomes that contain EEA1 was assessed in quadruplicate wells of *Mtb* infected BMDM by Immunofluorescence at 24hrs post-infection. (B) The percentage of *Mtb* phagosomes that were acidified was determined using LysoTracker staining of quadruplicate wells of infected cells at 24hrs post infection. (C) The percentage of *Mtb* containing phagosomes that contain V-ATPase was assessed in quadruplicate wells of *Mtb* infected BMDM by Immunofluorescence at 24hrs post-infection. ***p<0.0001 ANOVA Holm-Sidak post Hoc test. Data represents at least two independent experiments.
(TIFF)

S6 Fig SapM and PknG work in concert to arrest phagosome maturation. (A) The percentage of *Mtb* containing phagosomes that contain EEA1 was assessed in quadruplicate wells of *Mtb* infected BMDM by Immunofluorescence at 24hrs post-infection. (B)The percentage of *Mtb* phagosomes that were acidified was determined using LysoTracker staining of quadruplicate wells of infected cells at 24hrs post-infection. (C) The percentage of *Mtb* containing phagosomes that contain V-ATPase V_1_ B1/B2 was assessed in quadruplicate wells of *Mtb* infected BMDM by Immunofluorescence at 24hrs post-infection. (D) Triplicate wells of BMDM were infected at an MOI of 1 and CFU burden was assessed over the course of a 5 day infection. This graph portrays the entire time course corresponding to the data presented in Fig 5. ***p<0.0001 ANOVA Holm-Sidak post Hoc test. Data represents at least two independent experiments.
(TIFF)

S7 Fig SecA2 export is required to prevent autophagosome maturation. Quadruplicate wells of RAW-Difluo mLC3 cells were infected with H37Rv, the *secA2* mutant and the complemented strain at an MOI of 1. (A) The percentage of LC3 positive *Mtb* (RFP+) was assessed at 24hrs post infection. (B) The percentage of *Mtb* that was localized in an autophagolysosome (RFP+GFP-) was assessed at 24hrs post infection. Quadruplicate wells of RAW-Difluo mLC3 cells were infected with H37Rv and the *secA2* mutant SapM at an MOI of 1 One set of infected cells was treated with 100nm Bafilomycin A1 (Baf). (C) The percentage of LC3 positive *Mtb* (RFP+) was assessed at 1hr post infection. (D) The percentage of *Mtb* that was localized in an autophagolysosome (RFP+GFP-) was assessed at 1hr post infection. (E) Triplicate wells of RAW 264.7 cells were infected with H37Rv and the *secA2* mutant at an MOI of 1 and CFU burden was assessed over the course of a 5 day infection. ***p<0.0001 ANOVA Holm-Sidak post Hoc test. Data represents at least two independent experiments.
(TIFF)

S8 Fig SapM and PknG work in concert to arrest autophagosome maturation. Quadruplicate wells of RAW-Difluo mLC3 cells were infected with H37Rv and the *secA2* mutant SapM and/or PknG restoration strains at an MOI of 1. (A) The percentage of *Mtb* that was localized in an autophagolysosome (RFP+GFP-) was assessed at 24hrs post infection. (B) The percentage of *Mtb* phagosomes that were acidified was determined using LysoTracker (LT) staining of quadruplicate wells of infected cells at 24hrs post infection. Mature phagosomes were identified by lack of LC3 and presence of LT staining (LT+RFP-). (C and E) The percentage of *Mtb* that was localized in an autophagolysosome (RFP+GFP-) was assessed at both 1hr and 24hrs post infection. (D and F) The percentage of *Mtb* phagosomes that were acidified was determined using LysoTracker (LT) staining of quadruplicate wells of infected cells at both 1hr and 24hrs post infection. Mature phagosomes were identified by lack of LC3 (LC3-) and presence of LT staining (LT+RFP-). (G)The percentage of LC3+ *Mtb* (RFP+) was assessed at both 1hr and 24hrs post infection.*p<0.05 **p<0.001 ***p<0.0001 ANOVA Holm-Sidak post Hoc test. Data represents at least two independent experiments.
(TIFF)

S9 Fig NdkA and LpdC are exported in a SecA2 independent manner. Equal protein from cell lysates (WCL) or culture supernatants (Sup.) isolated from the wild-type strain H37Rv and the *secA2* mutant were examined for levels of NdkA or LpdC by Immunoblot.
(TIFF)

S10 Fig SecA2 is not required for *Mtb* resistance to oxidative stress. *Mtb* cultures were exposed to 5mM hydrogen peroxide for either 24 or 48 hours and then plated for viable CFU. Plotted is the percent survival relative to the starting inoculum. # indicates no viable CFU was recovered.
(TIFF)

S11 Fig *sapM* expression from a single-copy vector rescues defects of the *secA2* mutant in macrophages. (A)Phosphatase activity in triplicate culture supernatant samples was examined by quantifying cleavage of pNPP. Rates of pNPP cleavage were normalized to H37Rv. The relative phosphatase activity to H37Rv is indicated below the graph. (B) Triplicate wells of BMDM were infected at an MOI of 1 and CFU burden was assessed over the course of a 5 day infection. *p<0.001 ANOVA Holm-Sidak post Hoc test. Data represents at least two independent experiments. (C)The percentage of *Mtb* containing phagosomes that contain EEA1 was assessed in quadruplicate wells of *Mtb* infected BMDM by Immunofluorescence at 1hr post-infection. (D) The percentage of *Mtb* phagosomes that were acidified was determined using LysoTracker staining of quadruplicate wells of *Mtb* infected cells at 1hr post infection.
(TIFF)

S12 Fig Representative images of EEA1 immunofluorescent stained *Mtb* infected macrophages. *Mtb* infected macrophages were stained with antibodies to EEA1. Representative images of H37Rv and *secA2* mutant infected macrophages used to quantify co-localization are shown. *Mtb* autofluorescence was pseudo-colored green to highlight the co-localization in merged image.
(TIFF)

S13 Fig Representative images of Rab5 immunofluorescent stained *Mtb* infected macrophages. *Mtb* infected macrophages were stained with antibodies to Rab5. Representative images of H37Rv and *secA2* mutant infected macrophages used to quantify co-localization are shown. *Mtb* autofluorescence was pseudo-colored green to highlight the co-localization in merged image.
(TIFF)

S14 Fig Representative images of Rab7 immunofluorescent stained *Mtb* infected macrophages. *Mtb* infected macrophages were stained with antibodies to Rab7. Representative images of H37Rv and *secA2* mutant infected macrophages used to quantify co-localization are shown. *Mtb* autofluorescence was pseudo-colored green to highlight the co-localization in merged image.
(TIFF)

S15 Fig Representative images of V-ATPase V_1_ B1/B2 immunofluorescent stained *Mtb* infected macrophages. *Mtb* infected macrophages were stained with antibodies to V-ATPase V_1_ B1/B2. Representative images of H37Rv and *secA2* mutant infected macrophages used to quantify co-localization are shown. *Mtb* autofluorescence was pseudo-colored green to highlight the co-localization in merged image.
(TIFF)

S16 Fig Representative images of V-ATPase V_0_ A1 immunofluorescent stained *Mtb* infected macrophages. *Mtb* infected macrophages were stained with antibodies to V-ATPase V_0_ A1. Representative images of H37Rv and *secA2* mutant infected macrophages used to quantify co-localization are shown. *Mtb* autofluorescence was pseudo-colored green to highlight the co-localization in merged image.
(TIFF)

S17 Fig Representative images of Hv1 immunofluorescent stained *Mtb* infected macrophages. *Mtb* infected macrophages were stained with antibodies to Hv1. Representative images of H37Rv, Heat-killed H37Rv and *M*. *smegmatis* infected macrophages used to quantify co-localization are shown. Mycobacterial autofluorescence was pseudo-colored green to highlight the co-localization in merged image.
(TIFF)

S18 Fig Representative images of Lysotracker stained *Mtb* infected macrophages. *Mtb* infected macrophages were stained Lysotracker. Representative images of H37Rv and *secA2* mutant infected macrophages used to quantify co-localization are shown. *Mtb* autofluorescence was pseudo-colored green to highlight the co-localization in merged image.
(TIFF)

S19 Fig Representative images of Lysotracker stained *Mtb* infected RAW-Difluo mLC3 cells. *Mtb* infected RAW-Difluo mLC3 cells were stained with Lysotracker. Representative images of H37Rv and *secA2* mutant infected macrophages used to quantify co-localization are shown.
(TIFF)
