# Supplementary figures and images for: The SecA2 pathway of Mycobacterium tuberculosis exports effectors that work in concert to arrest phagosome and autophagosome maturation
Source: PLoS Pathog. 2018 Apr 30;14(4):e1007011. doi: 10.1371/journal.ppat.1007011 (PMC5945054; doi:10.1371/journal.ppat.1007011)

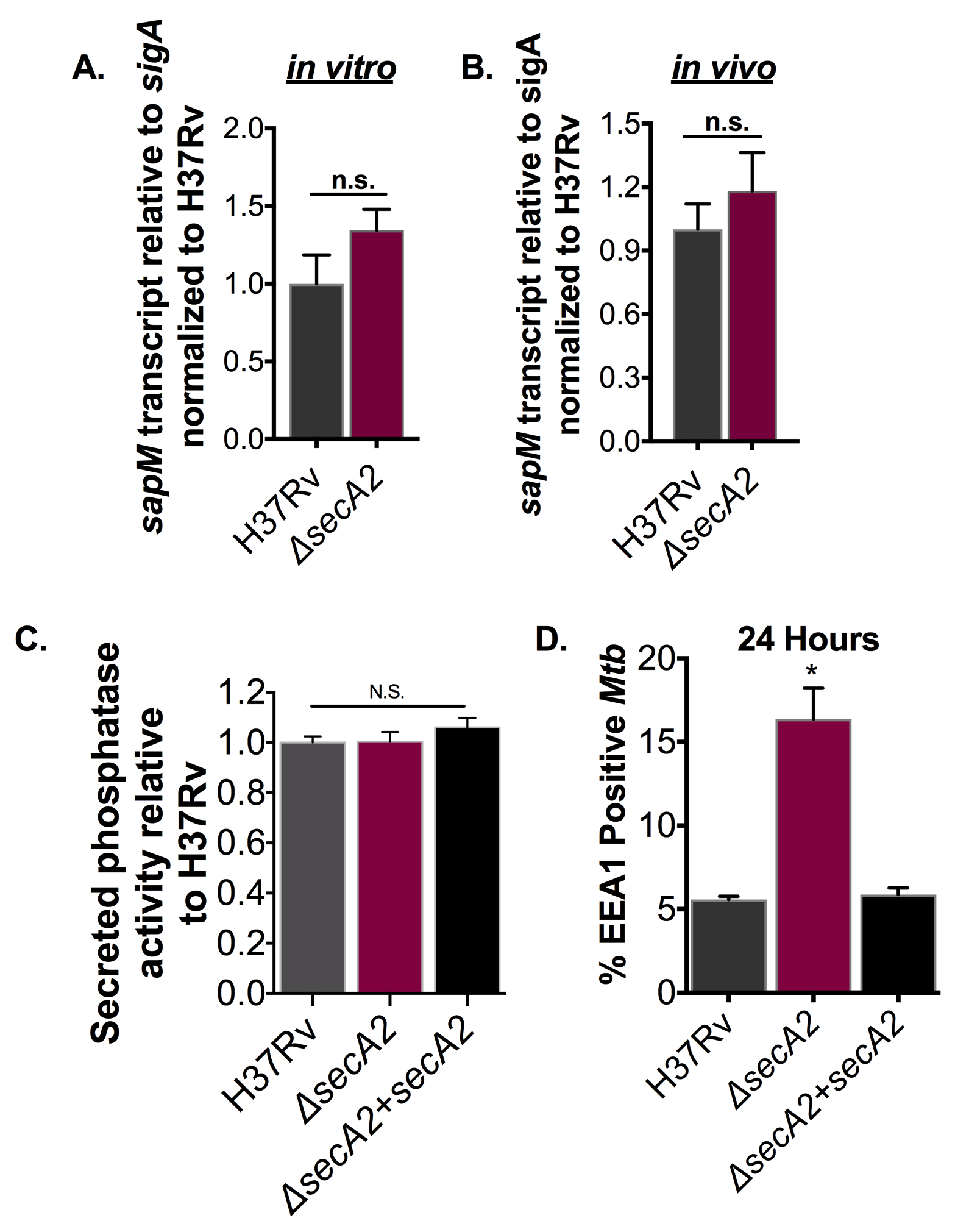

Supplement: S1 Fig — (A)RNA was isolated from triplicate broth cultures of H37Rv and the secA2 mutant and sapM transcript was quantified by RT-PCR. Transcripts were normalized to the housekeeping gene sigA. (B)RNA was isolated from triplicate samples of RAW 264.7 cells infected with either H37Rv or the secA2 mutant and sapM transcript was quantified by RT-PCR. Transcripts were normalized to the housekeeping gene sigA. (C) Phosphatase activity in triplicate culture supernatant samples was examined by quantifying cleavage of pNPP in the presence of sodium molybdate. Rates of pNPP cleavage were normalized to H37Rv. (D) The percentage of Mtb containing phagosomes that contain EEA1 was assessed in quadruplicate wells of Mtb infected BMDM by Immunofluorescence at 24hrs post-infection. *p<0.001 ANOVA Holm-Sidak post Hoc test. Data represents at least two independent experiments. (TIFF) [file ppat.1007011.s003.tiff]

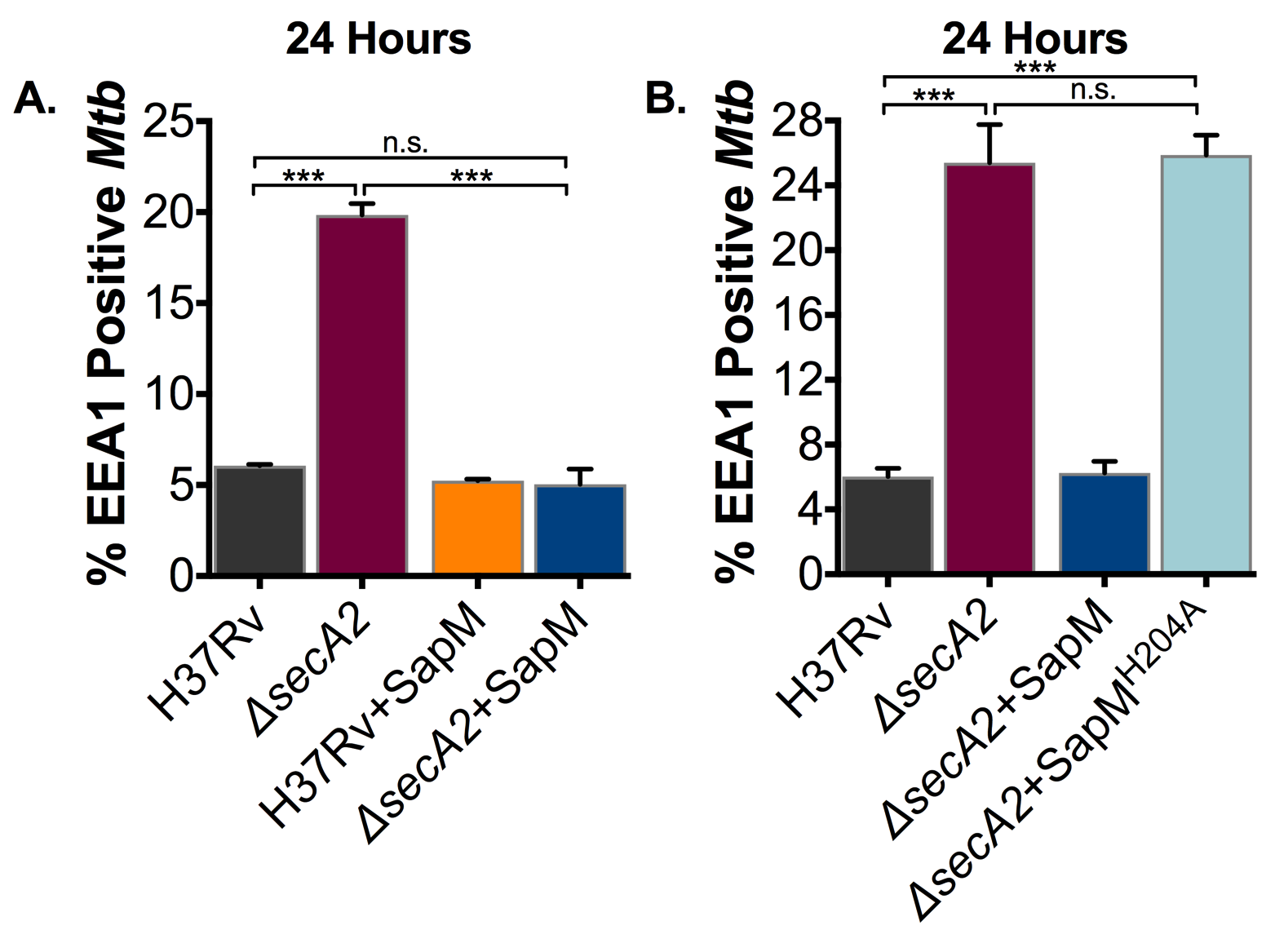

Supplement: S2 Fig — (A and B) The percentage of Mtb containing phagosomes that contain EEA1 was assessed in quadruplicate wells of Mtb infected BMDM by Immunofluorescence at 24hrs post-infection. ***p<0.0001 ANOVA Holm-Sidak post Hoc test. Data represents at least two independent experiments. (TIFF) [file ppat.1007011.s004.tiff]

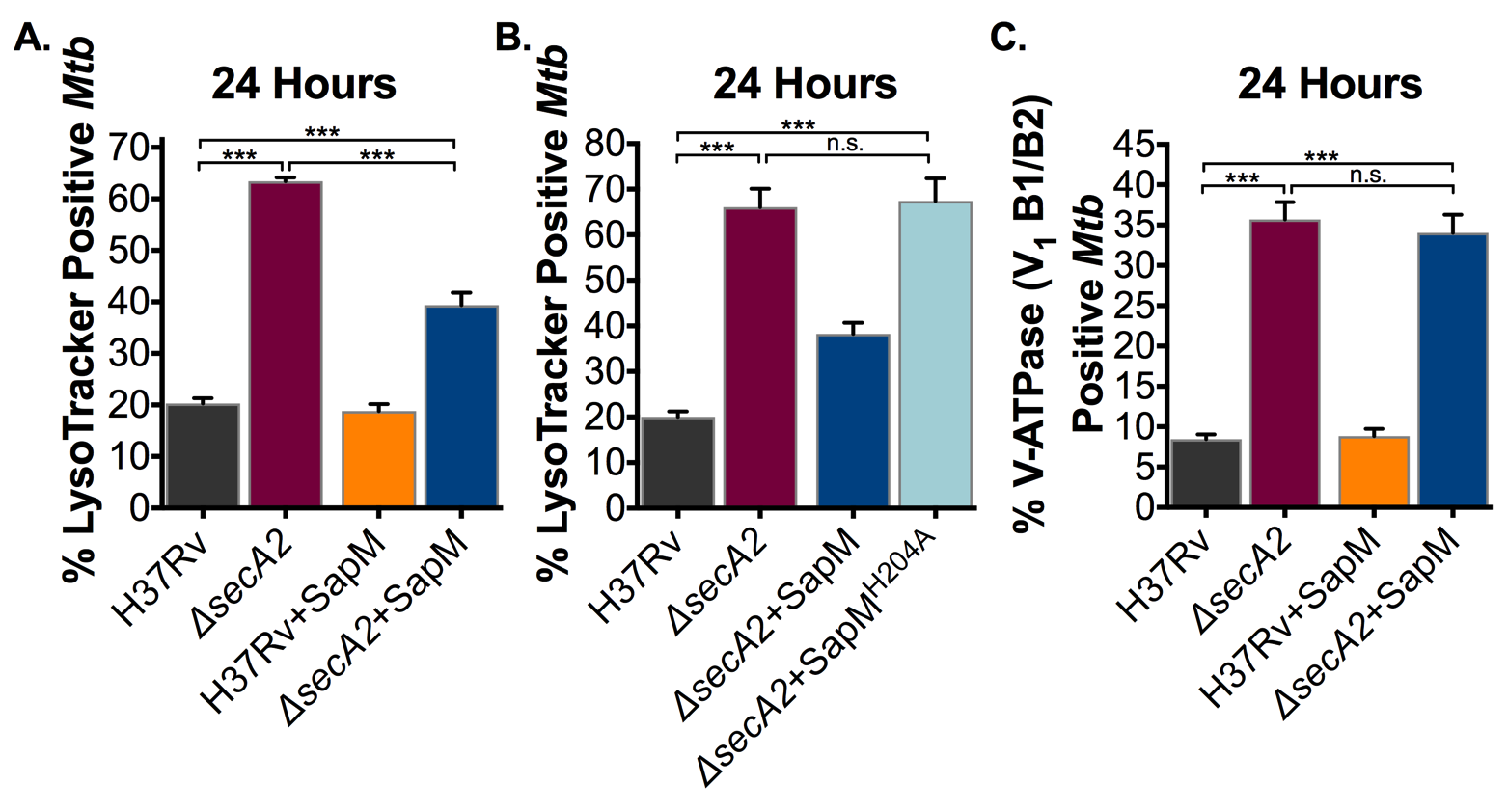

Supplement: S3 Fig — (A and B) The percentage of Mtb phagosomes that were acidified was determined using LysoTracker staining of quadruplicate wells of infected cells at 24hrs post infection (C) The percentage of Mtb containing phagosomes that contain V-ATPase was assessed in quadruplicate wells of Mtb infected BMDM by Immunofluorescence at 24hrs post infection. ***p<0.0001 ANOVA Holm-Sidak post Hoc test. Data represents at least two independent experiments. (TIFF) [file ppat.1007011.s005.tiff]

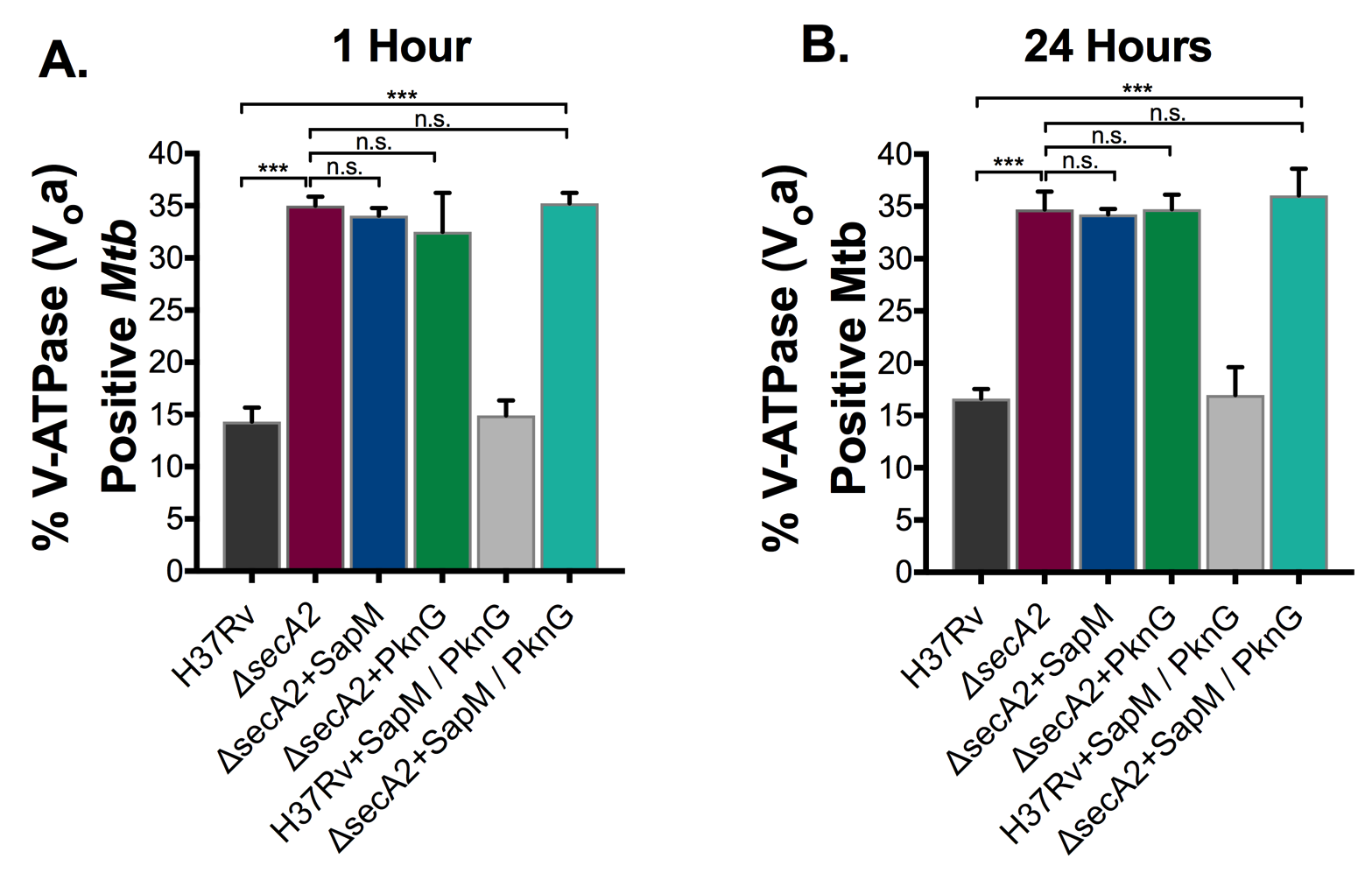

Supplement: S4 Fig — The percentage of Mtb containing phagosomes that contain V-ATPase subunit V0 a1 was assessed in quadruplicate wells of Mtb infected BMDM by Immunofluorescence at (A) 1hr and (B) 24 hours post-infection. *p<0.05 **p<0.001 ***p<0.0001 ANOVA Holm-Sidak post Hoc test. Data represents at least two independent experiments. (TIFF) [file ppat.1007011.s006.tiff]

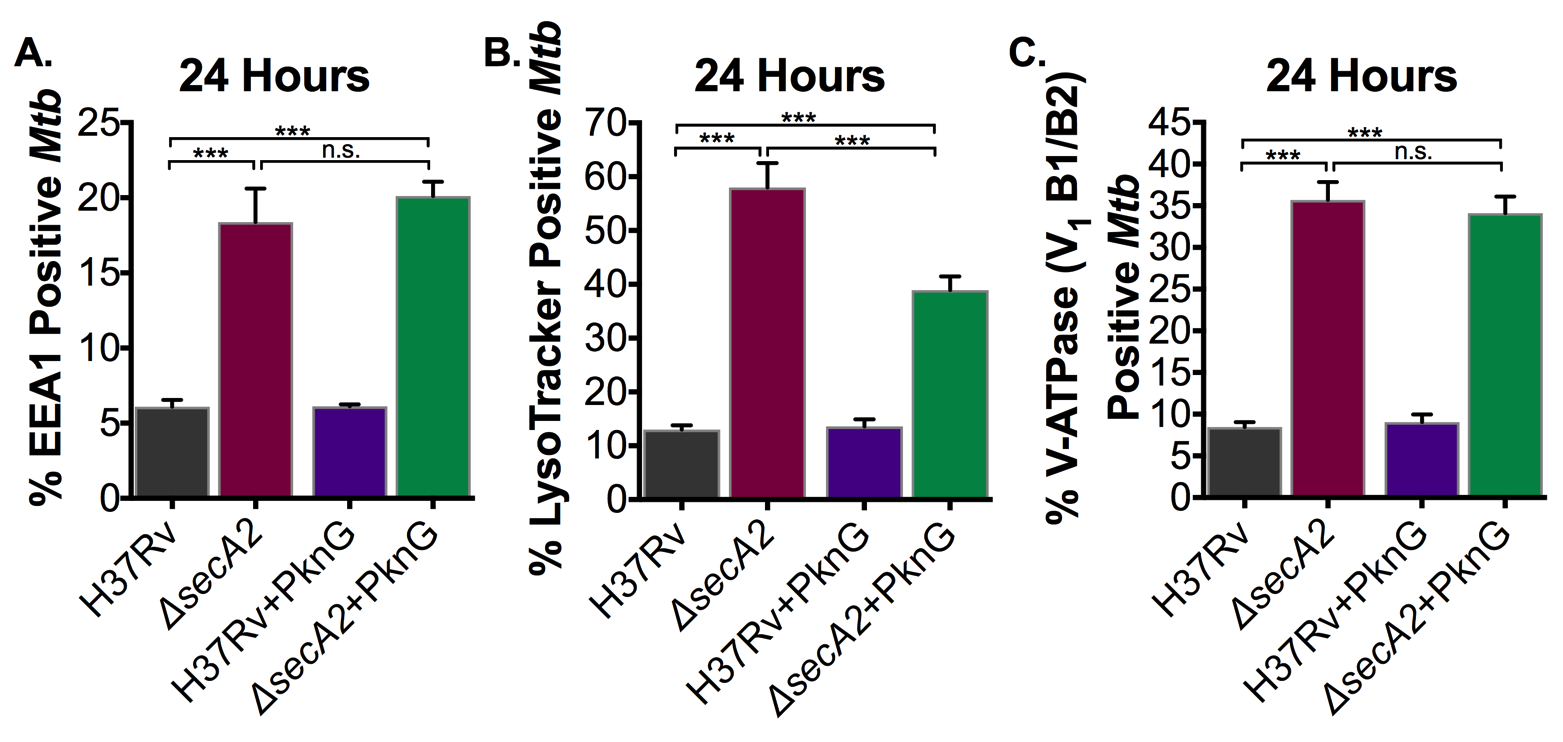

Supplement: S5 Fig — (A) The percentage of Mtb containing phagosomes that contain EEA1 was assessed in quadruplicate wells of Mtb infected BMDM by Immunofluorescence at 24hrs post-infection. (B) The percentage of Mtb phagosomes that were acidified was determined using LysoTracker staining of quadruplicate wells of infected cells at 24hrs post infection. (C) The percentage of Mtb containing phagosomes that contain V-ATPase was assessed in quadruplicate wells of Mtb infected BMDM by Immunofluorescence at 24hrs post-infection. ***p<0.0001 ANOVA Holm-Sidak post Hoc test. Data represents at least two independent experiments. (TIFF) [file ppat.1007011.s007.tiff]

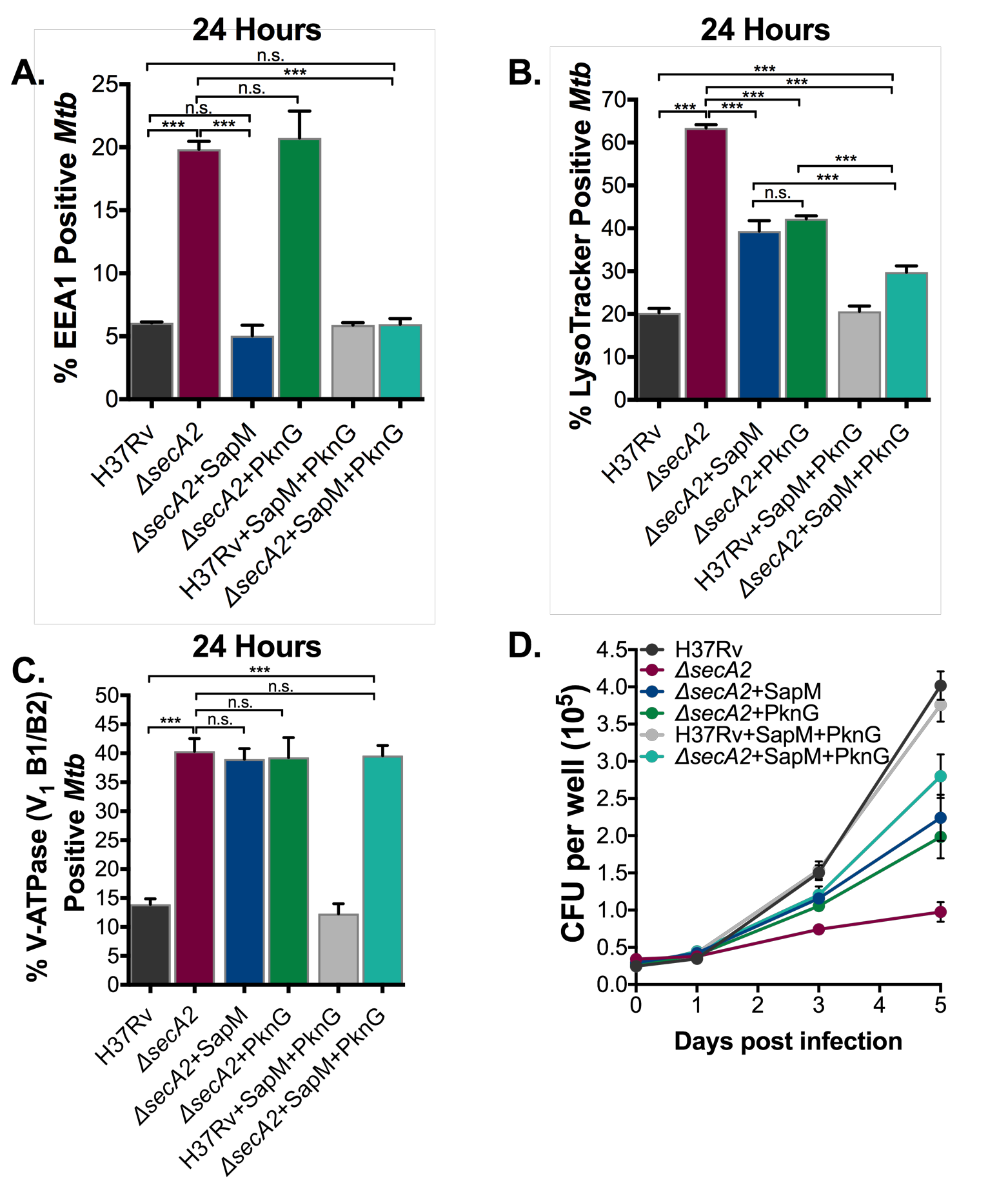

Supplement: S6 Fig — (A) The percentage of Mtb containing phagosomes that contain EEA1 was assessed in quadruplicate wells of Mtb infected BMDM by Immunofluorescence at 24hrs post-infection. (B)The percentage of Mtb phagosomes that were acidified was determined using LysoTracker staining of quadruplicate wells of infected cells at 24hrs post-infection. (C) The percentage of Mtb containing phagosomes that contain V-ATPase V1 B1/B2 was assessed in quadruplicate wells of Mtb infected BMDM by Immunofluorescence at 24hrs post-infection. (D) Triplicate wells of BMDM were infected at an MOI of 1 and CFU burden was assessed over the course of a 5 day infection. This graph portrays the entire time course corresponding to the data presented in Fig 5. ***p<0.0001 ANOVA Holm-Sidak post Hoc test. Data represents at least two independent experiments. (TIFF) [file ppat.1007011.s008.tiff]

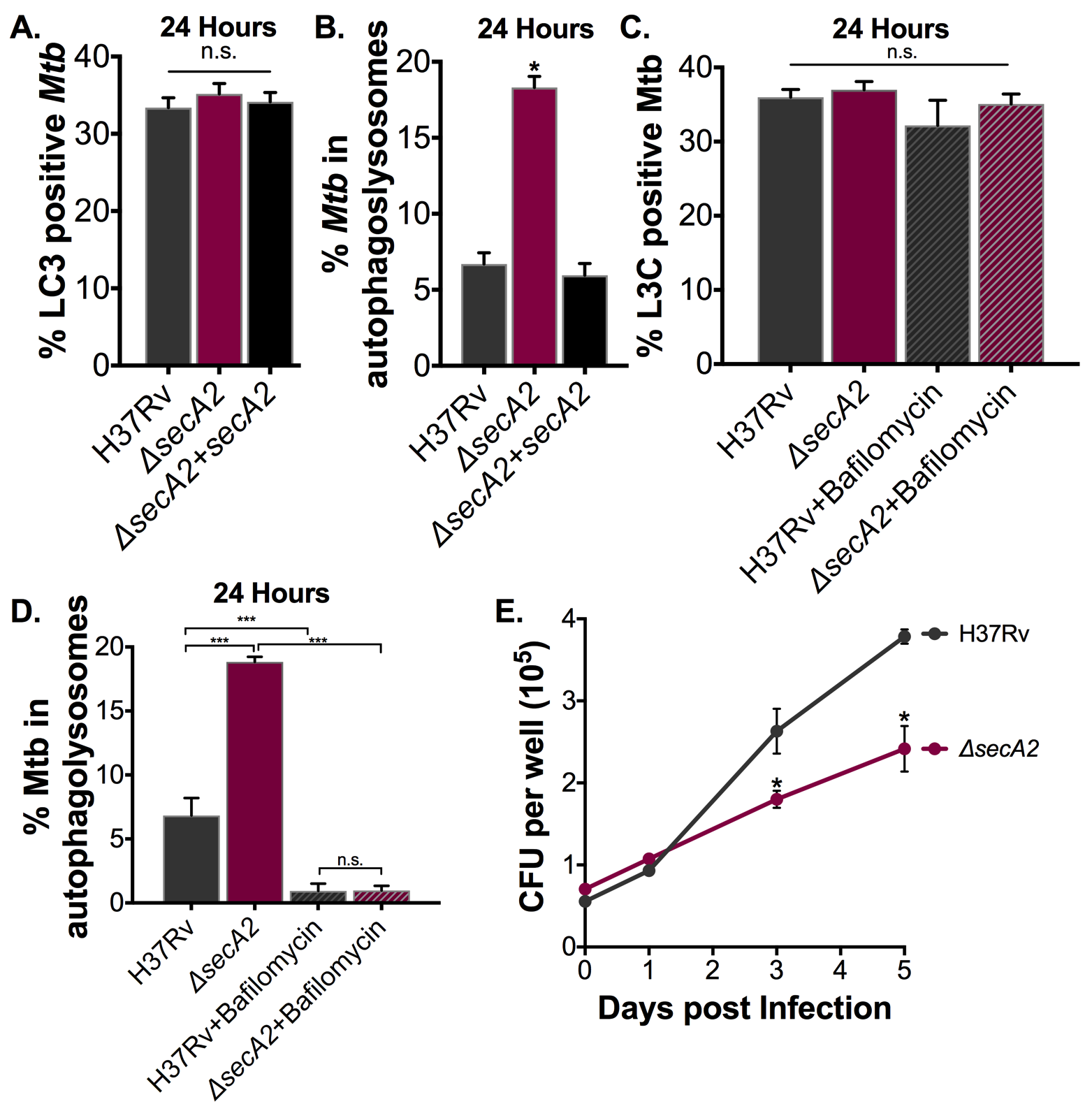

Supplement: S7 Fig — Quadruplicate wells of RAW-Difluo mLC3 cells were infected with H37Rv, the secA2 mutant and the complemented strain at an MOI of 1. (A) The percentage of LC3 positive Mtb (RFP+) was assessed at 24hrs post infection. (B) The percentage of Mtb that was localized in an autophagolysosome (RFP+GFP-) was assessed at 24hrs post infection. Quadruplicate wells of RAW-Difluo mLC3 cells were infected with H37Rv and the secA2 mutant SapM at an MOI of 1 One set of infected cells was treated with 100nm Bafilomycin A1 (Baf). (C) The percentage of LC3 positive Mtb (RFP+) was assessed at 1hr post infection. (D) The percentage of Mtb that was localized in an autophagolysosome (RFP+GFP-) was assessed at 1hr post infection. (E) Triplicate wells of RAW 264.7 cells were infected with H37Rv and the secA2 mutant at an MOI of 1 and CFU burden was assessed over the course of a 5 day infection. ***p<0.0001 ANOVA Holm-Sidak post Hoc test. Data represents at least two independent experiments. (TIFF) [file ppat.1007011.s009.tiff]

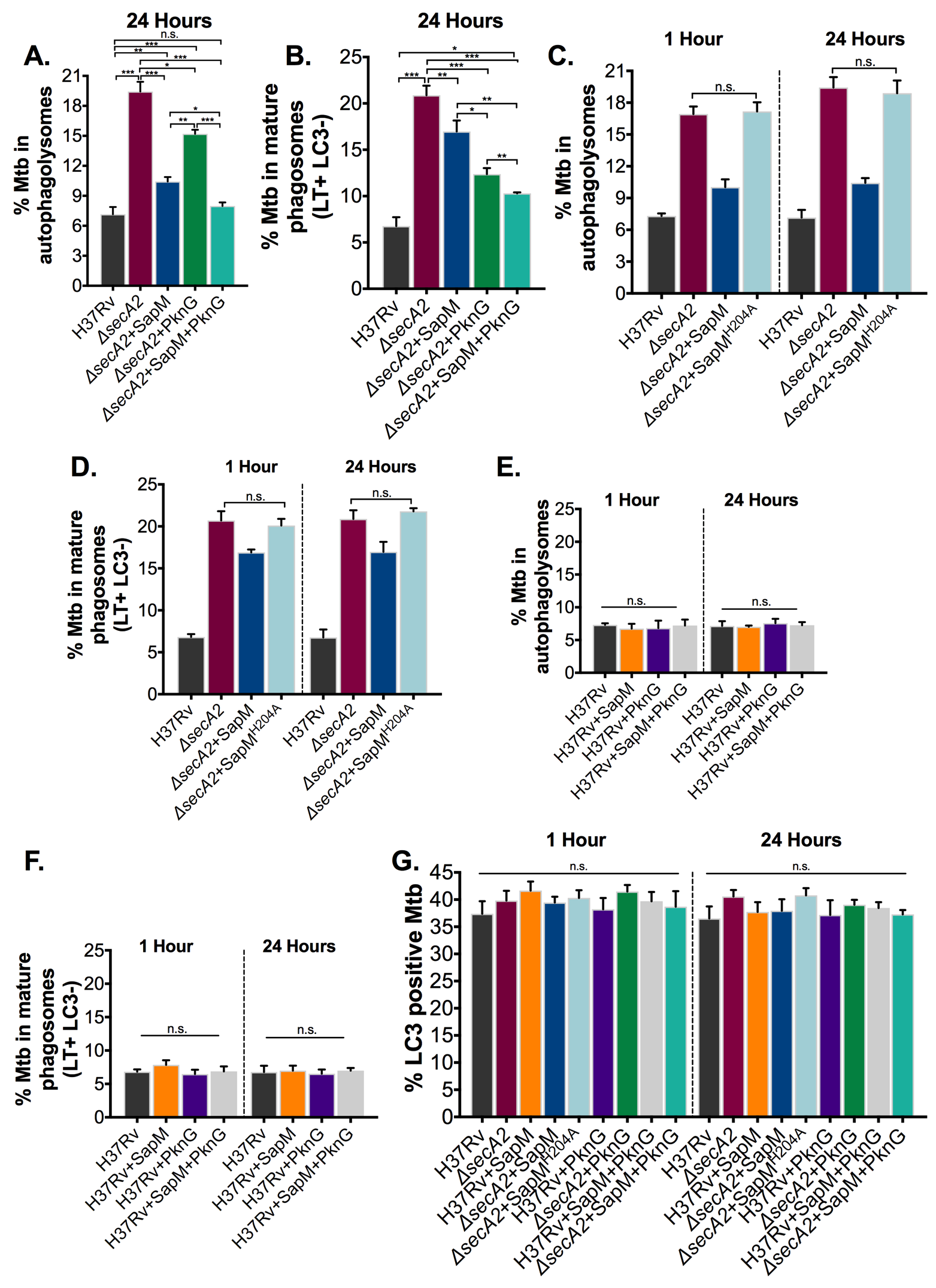

Supplement: S8 Fig — Quadruplicate wells of RAW-Difluo mLC3 cells were infected with H37Rv and the secA2 mutant SapM and/or PknG restoration strains at an MOI of 1. (A) The percentage of Mtb that was localized in an autophagolysosome (RFP+GFP-) was assessed at 24hrs post infection. (B) The percentage of Mtb phagosomes that were acidified was determined using LysoTracker (LT) staining of quadruplicate wells of infected cells at 24hrs post infection. Mature phagosomes were identified by lack of LC3 and presence of LT staining (LT+RFP-). (C and E) The percentage of Mtb that was localized in an autophagolysosome (RFP+GFP-) was assessed at both 1hr and 24hrs post infection. (D and F) The percentage of Mtb phagosomes that were acidified was determined using LysoTracker (LT) staining of quadruplicate wells of infected cells at both 1hr and 24hrs post infection. Mature phagosomes were identified by lack of LC3 (LC3-) and presence of LT staining (LT+RFP-). (G)The percentage of LC3+ Mtb (RFP+) was assessed at both 1hr and 24hrs post infection.*p<0.05 **p<0.001 ***p<0.0001 ANOVA Holm-Sidak post Hoc test. Data represents at least two independent experiments. (TIFF) [file ppat.1007011.s010.tiff]

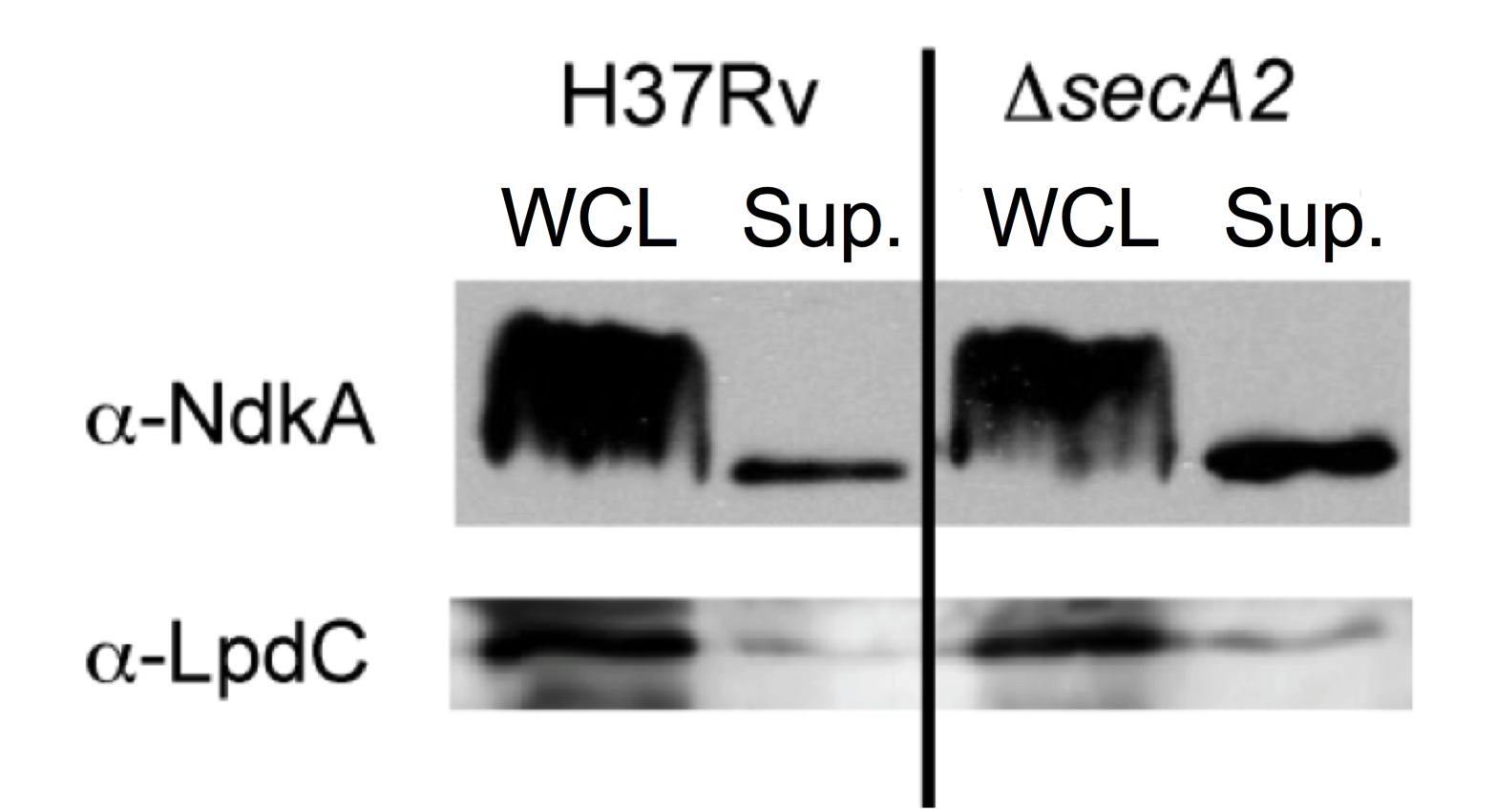

Supplement: S9 Fig — Equal protein from cell lysates (WCL) or culture supernatants (Sup.) isolated from the wild-type strain H37Rv and the secA2 mutant were examined for levels of NdkA or LpdC by Immunoblot. (TIFF) [file ppat.1007011.s011.tiff]

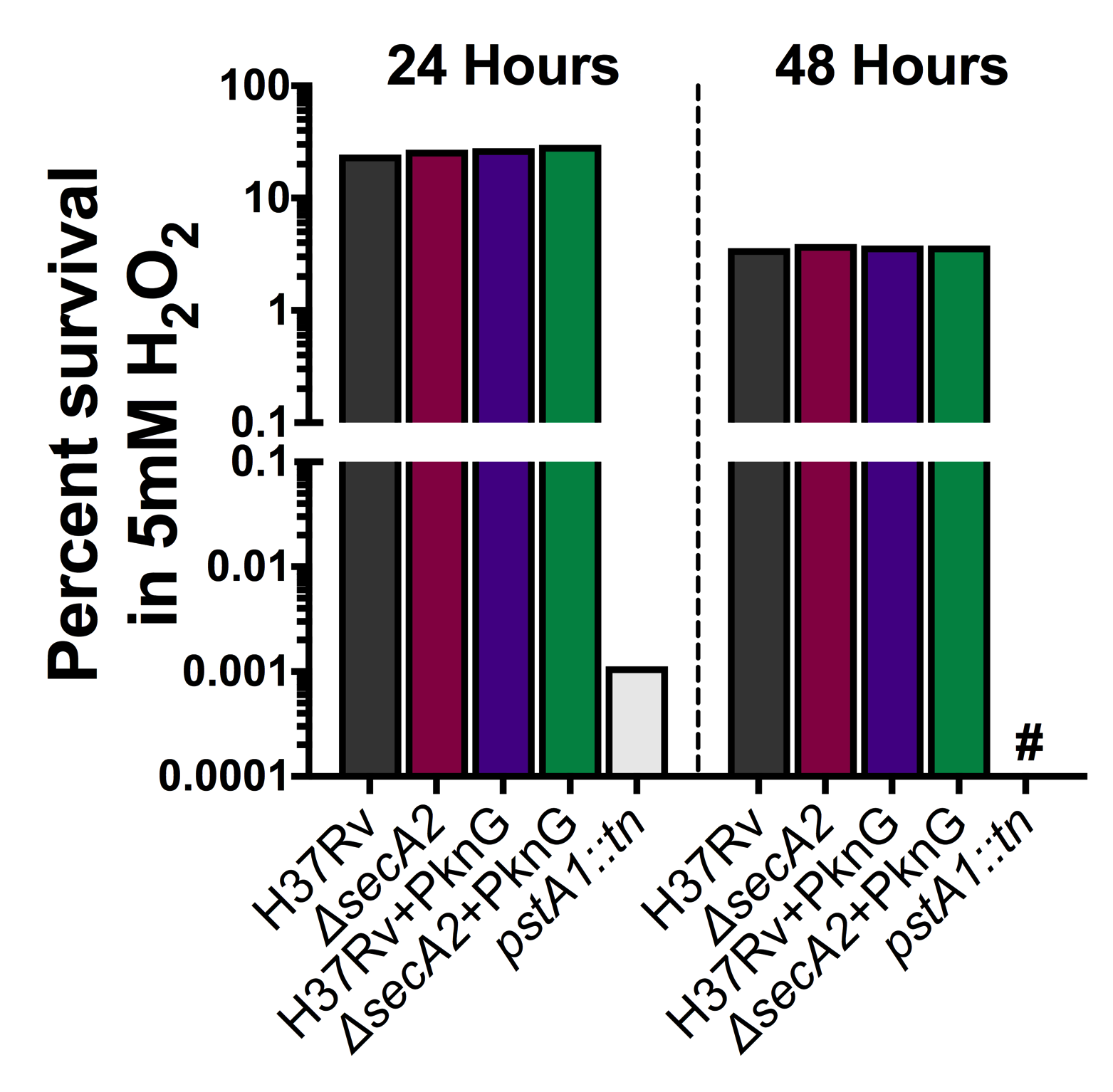

Supplement: S10 Fig — Mtb cultures were exposed to 5mM hydrogen peroxide for either 24 or 48 hours and then plated for viable CFU. Plotted is the percent survival relative to the starting inoculum. # indicates no viable CFU was recovered. (TIFF) [file ppat.1007011.s012.tiff]

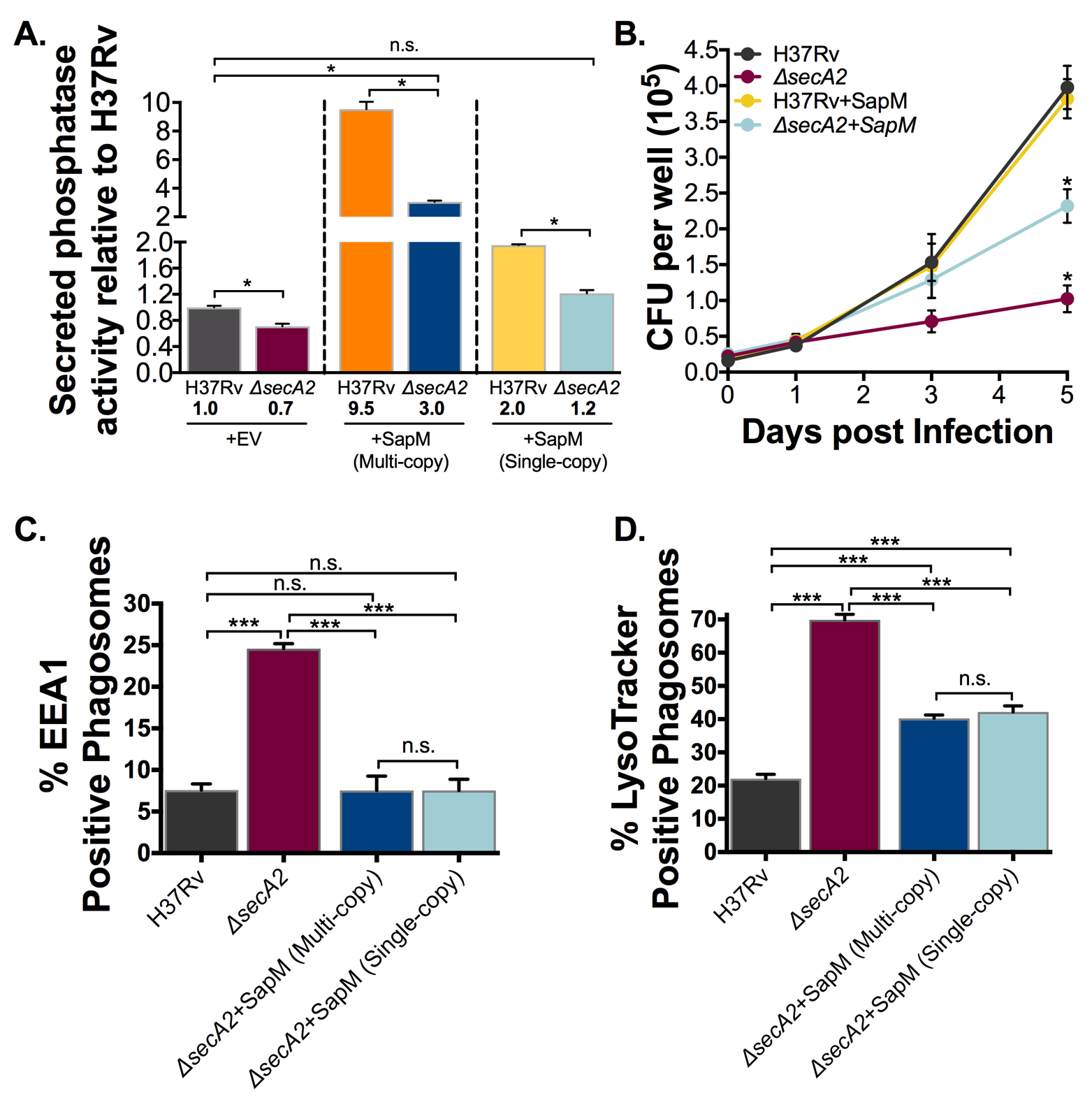

Supplement: S11 Fig — (A)Phosphatase activity in triplicate culture supernatant samples was examined by quantifying cleavage of pNPP. Rates of pNPP cleavage were normalized to H37Rv. The relative phosphatase activity to H37Rv is indicated below the graph. (B) Triplicate wells of BMDM were infected at an MOI of 1 and CFU burden was assessed over the course of a 5 day infection. *p<0.001 ANOVA Holm-Sidak post Hoc test. Data represents at least two independent experiments. (C)The percentage of Mtb containing phagosomes that contain EEA1 was assessed in quadruplicate wells of Mtb infected BMDM by Immunofluorescence at 1hr post-infection. (D) The percentage of Mtb phagosomes that were acidified was determined using LysoTracker staining of quadruplicate wells of Mtb infected cells at 1hr post infection. (TIFF) [file ppat.1007011.s013.tiff]

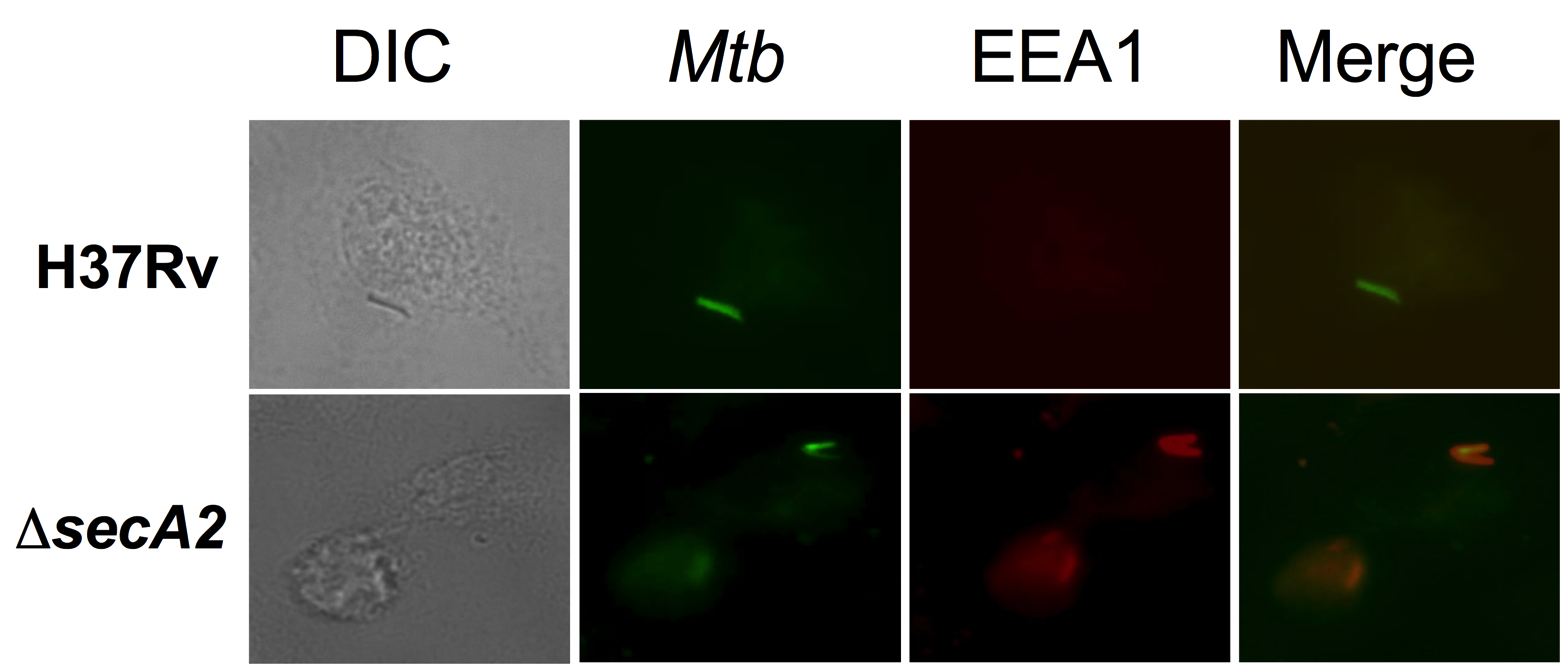

Supplement: S12 Fig — Mtb infected macrophages were stained with antibodies to EEA1. Representative images of H37Rv and secA2 mutant infected macrophages used to quantify co-localization are shown. Mtb autofluorescence was pseudo-colored green to highlight the co-localization in merged image. (TIFF) [file ppat.1007011.s014.tiff]

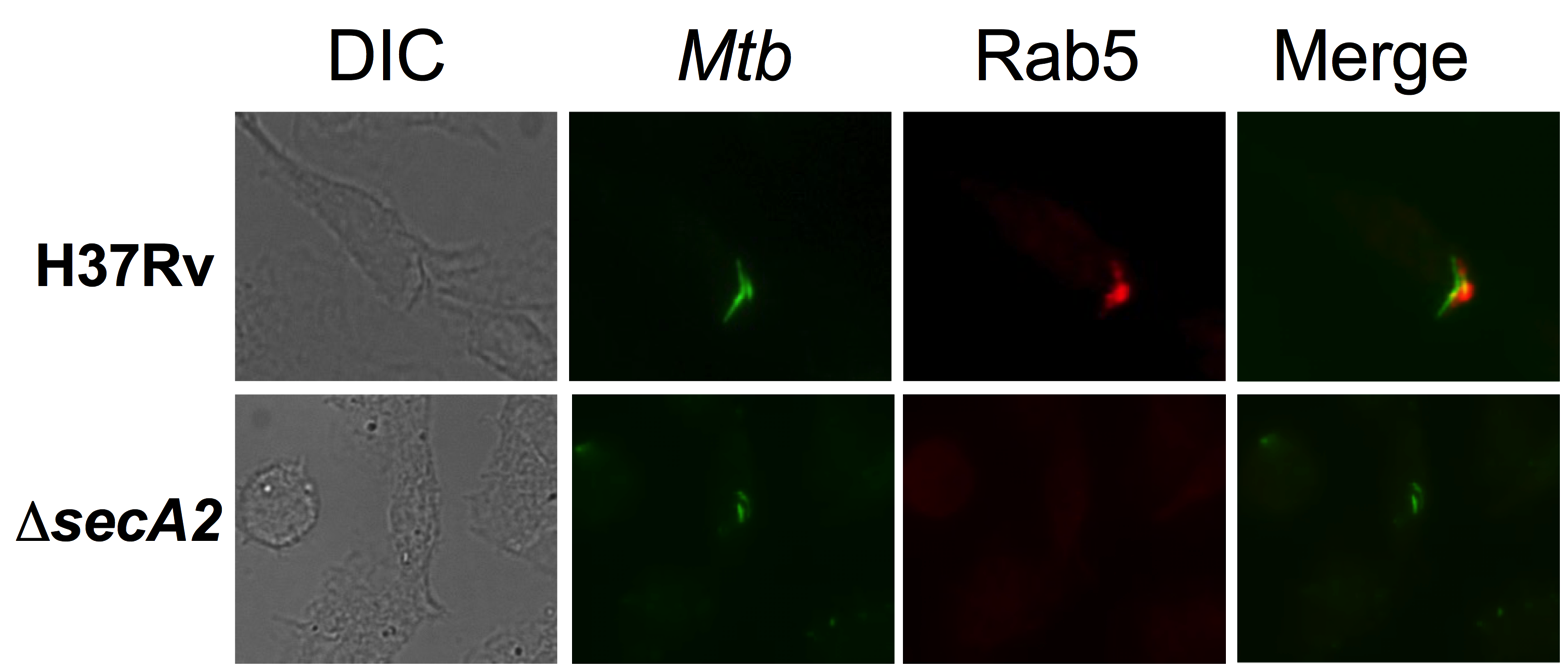

Supplement: S13 Fig — Mtb infected macrophages were stained with antibodies to Rab5. Representative images of H37Rv and secA2 mutant infected macrophages used to quantify co-localization are shown. Mtb autofluorescence was pseudo-colored green to highlight the co-localization in merged image. (TIFF) [file ppat.1007011.s015.tiff]

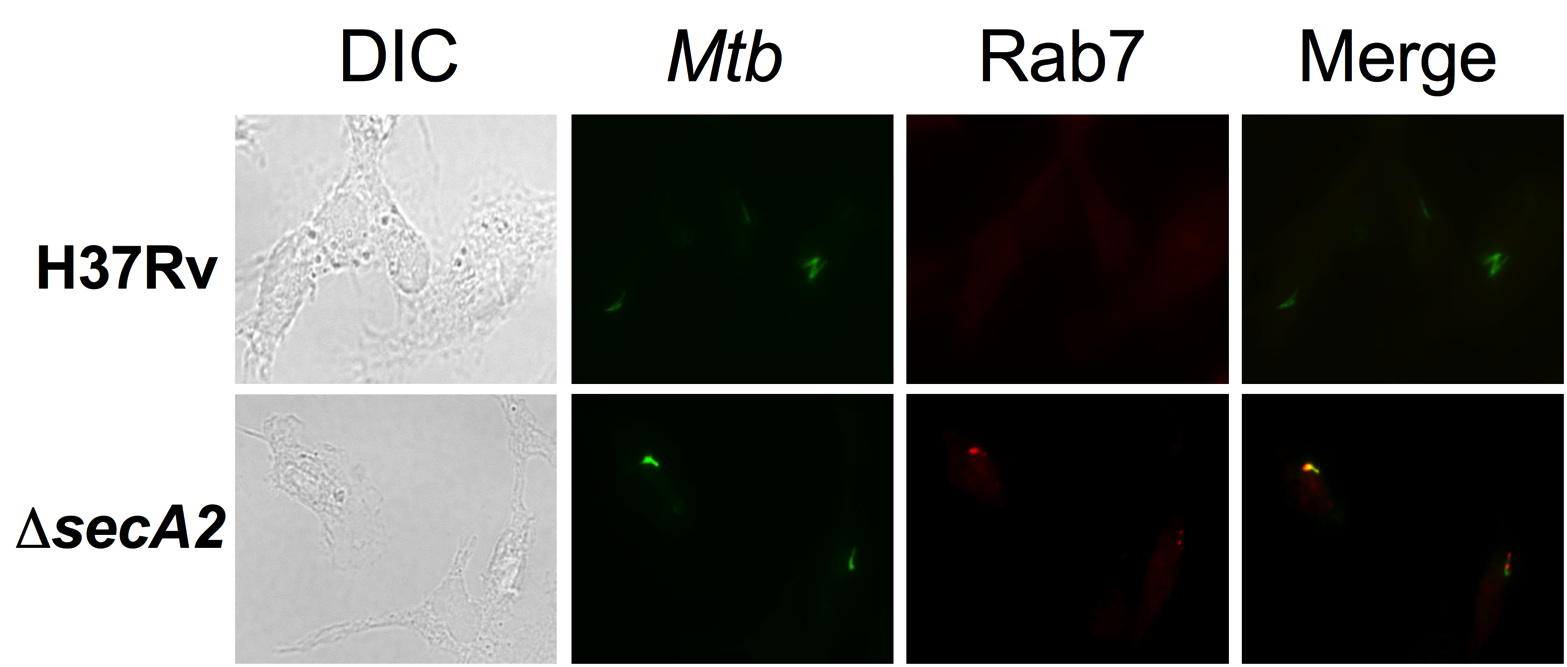

Supplement: S14 Fig — Mtb infected macrophages were stained with antibodies to Rab7. Representative images of H37Rv and secA2 mutant infected macrophages used to quantify co-localization are shown. Mtb autofluorescence was pseudo-colored green to highlight the co-localization in merged image. (TIFF) [file ppat.1007011.s016.tiff]

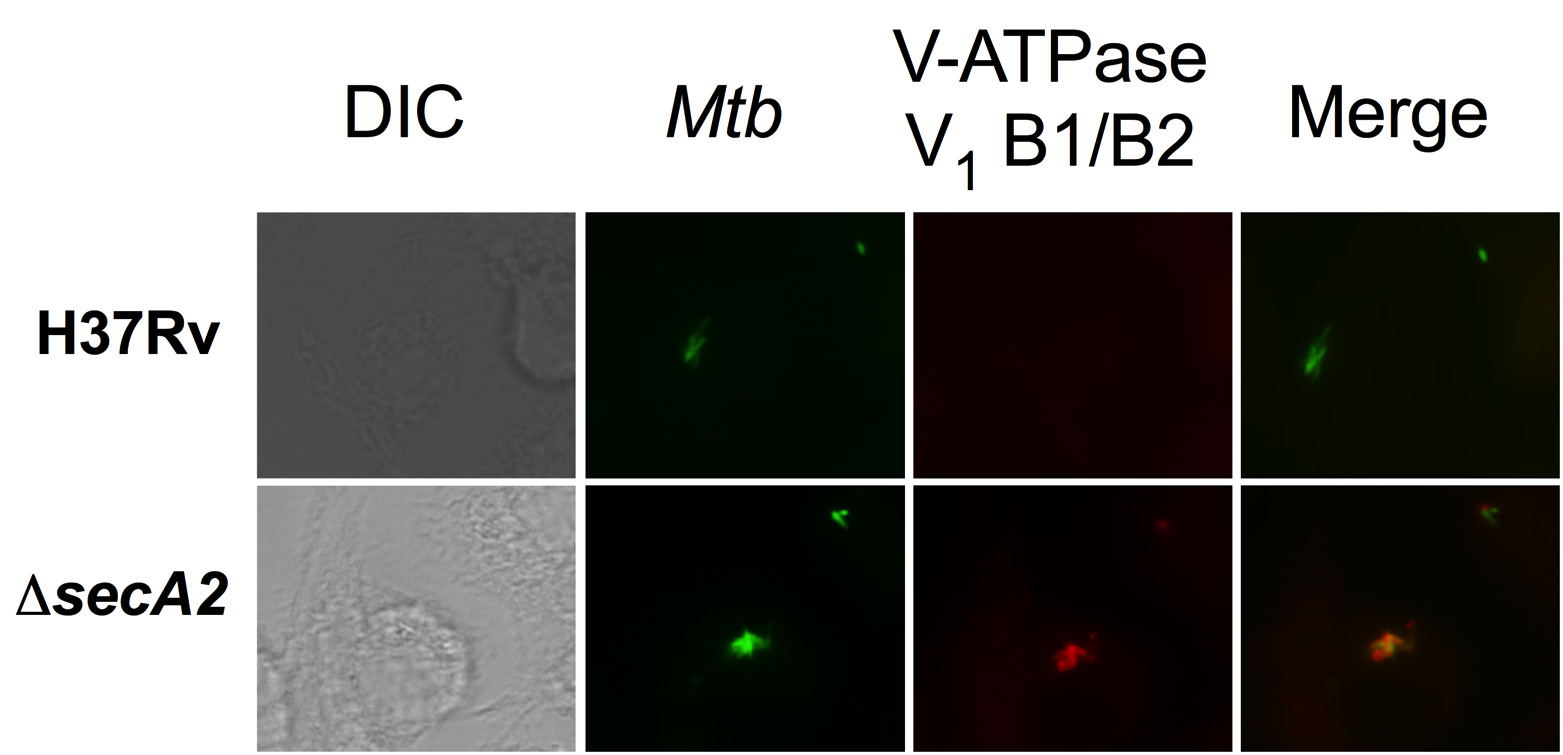

Supplement: S15 Fig — Mtb infected macrophages were stained with antibodies to V-ATPase V1 B1/B2. Representative images of H37Rv and secA2 mutant infected macrophages used to quantify co-localization are shown. Mtb autofluorescence was pseudo-colored green to highlight the co-localization in merged image. (TIFF) [file ppat.1007011.s017.tiff]

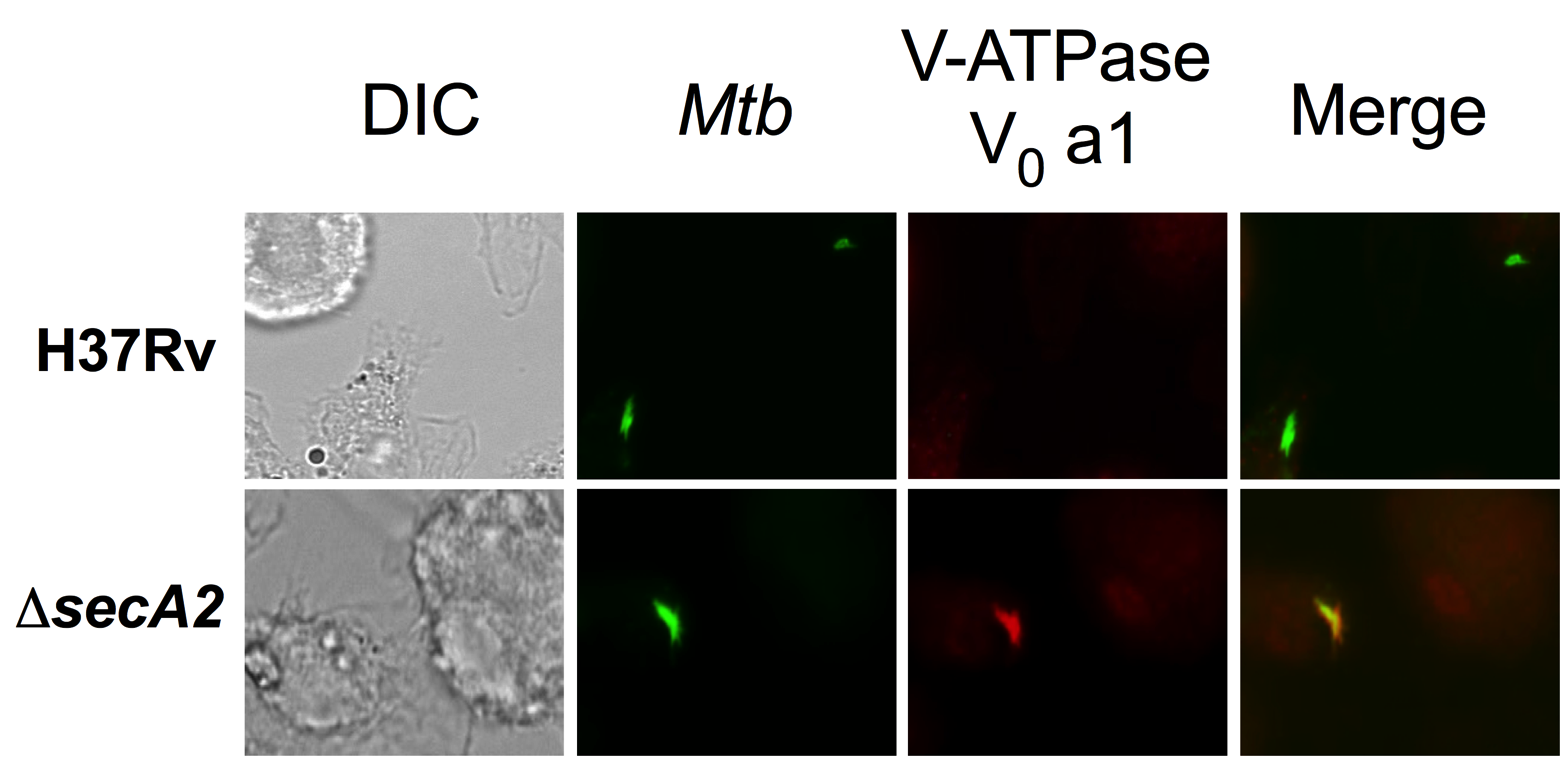

Supplement: S16 Fig — Mtb infected macrophages were stained with antibodies to V-ATPase V0 A1. Representative images of H37Rv and secA2 mutant infected macrophages used to quantify co-localization are shown. Mtb autofluorescence was pseudo-colored green to highlight the co-localization in merged image. (TIFF) [file ppat.1007011.s018.tiff]

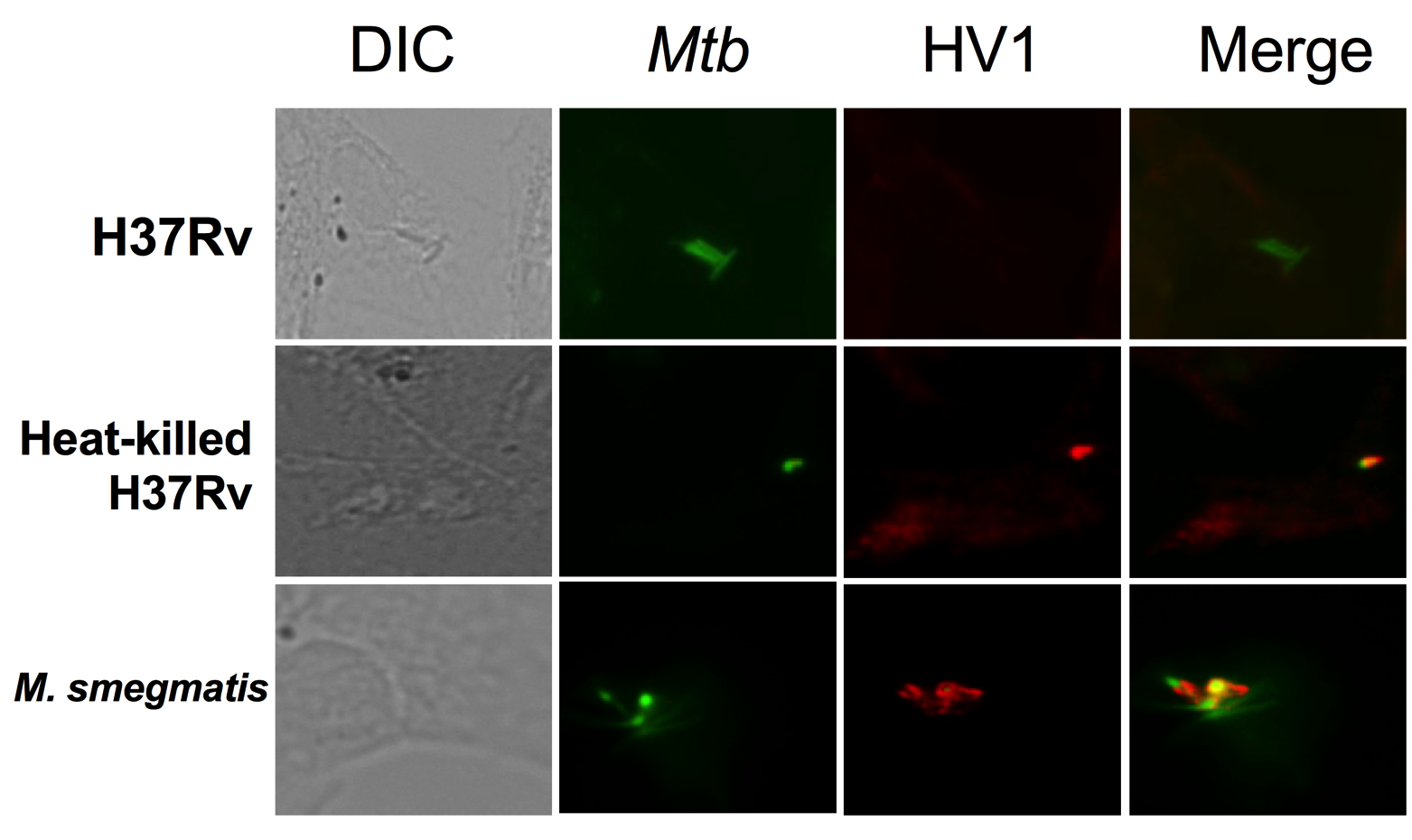

Supplement: S17 Fig — Mtb infected macrophages were stained with antibodies to Hv1. Representative images of H37Rv, Heat-killed H37Rv and M. smegmatis infected macrophages used to quantify co-localization are shown. Mycobacterial autofluorescence was pseudo-colored green to highlight the co-localization in merged image. (TIFF) [file ppat.1007011.s019.tiff]

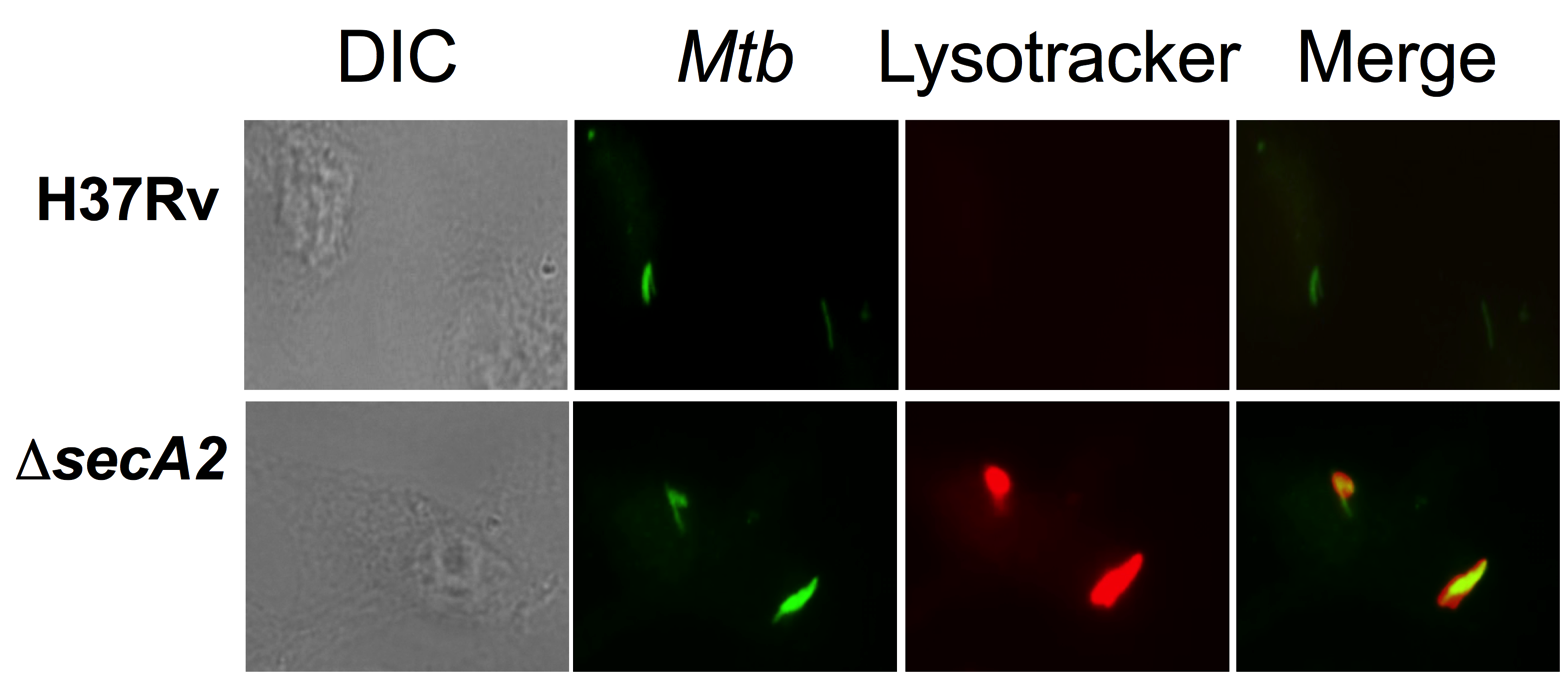

Supplement: S18 Fig — Mtb infected macrophages were stained Lysotracker. Representative images of H37Rv and secA2 mutant infected macrophages used to quantify co-localization are shown. Mtb autofluorescence was pseudo-colored green to highlight the co-localization in merged image. (TIFF) [file ppat.1007011.s020.tiff]

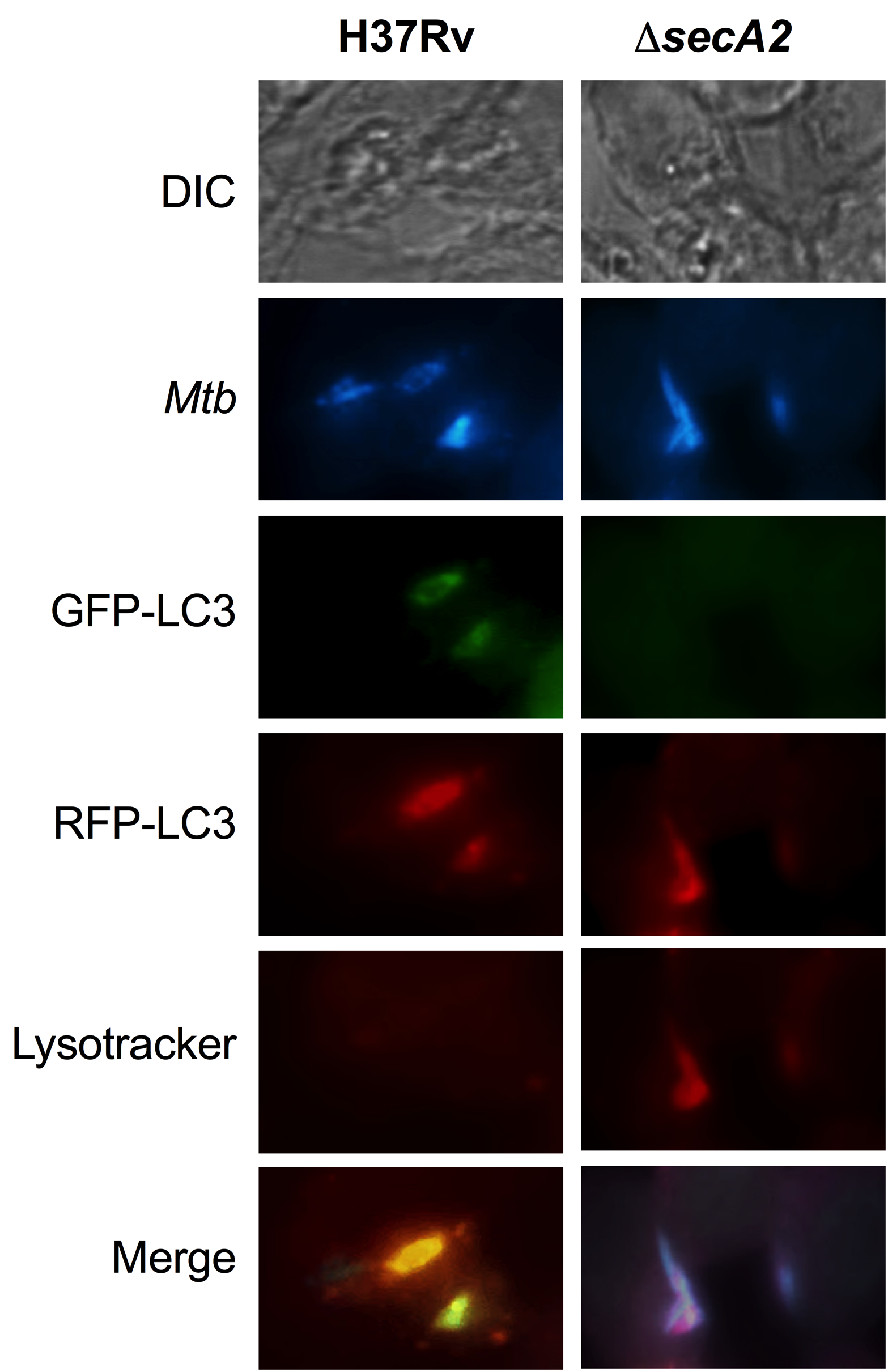

Supplement: S19 Fig — Mtb infected RAW-Difluo mLC3 cells were stained with Lysotracker. Representative images of H37Rv and secA2 mutant infected macrophages used to quantify co-localization are shown. (TIFF) [file ppat.1007011.s021.tiff]
